# Colonoid-Based Transcriptomics Reveals Conserved and Model-Specific Mechanisms of Doxorubicin-Induced Intestinal Toxicity

**DOI:** 10.3390/ijms27167072

**Published:** 2026-08-07

**Authors:** Saad Lodhi, Marcel Van Herwijnen, Colette Kelly, Harvey Fowler-Williams, Carrie A. Duckworth, D. Mark Pritchard, Florian Caiment, Theo M. C. M. de Kok, Marcha C. T. Verheijen, Danyel G. J. Jennen

**Affiliations:** 1 Department of Translational Genomics, GROW—Research Institute for Oncology and Reproduction, Maastricht University, 6229 ER Maastricht, The Netherlandsdanyel.jennen@maastrichtuniversity.nl (D.G.J.J.); 2 Department of Molecular and Clinical Cancer Medicine, Institute of Systems, Molecular and Integrative Biology, University of Liverpool, Liverpool L69 3GE, UK

**Keywords:** gastrointestinal toxicity, doxorubicin, intestinal organoids, transcriptomics, new approach methodologies

## Abstract

Animal models are standard for safety evaluation, yet physiological differences limit human translation. Doxorubicin, a chemotherapeutic agent, can cause off-target gastrointestinal toxicity and treatment discontinuation. This study evaluated human colonoids as a controlled human-derived epithelial model for doxorubicin-induced gastrointestinal toxicity by comparing transcriptomic responses across human colonoids, mouse colonoids, and male C57BL/6J mouse colon tissue. Within each dataset, doxorubicin-treated conditions were pooled across model-specific exposure levels and time points to estimate broad doxorubicin-associated transcriptional signatures. Using a parallelogram approach, differential expression, co-expression, pathway mapping, and Comparative Toxicogenomics Database benchmarking were applied to compare model concordance and identify doxorubicin-responsive mechanisms. Shared responses converged on cell-cycle regulation, DNA damage response, DNA repair, and apoptosis. Concordance in differentially expressed genes was highest between mouse colonoids and mouse colon, while pathway mapping showed similarities between colonoid systems. A core set of p53-associated genes, including *BAX*, *INKA2*, and *ZMAT3*, was shared across datasets, with additional apoptotic and DNA damage response features observed in colonoids. Comparative Toxicogenomics Database benchmarking supported doxorubicin biology and highlighted underrepresented gastrointestinal toxicity-relevant signals, indicating gaps in intestinal toxicogenomic annotations. Colonoid-based transcriptomics supports human colonoids as controlled epithelial models for mechanistic gastrointestinal toxicity assessment, although missing vascular, immune, and systemic context means clinical validation remains necessary.

## 1. Introduction

Animal models have been used consistently to assess drug toxicity and efficacy [1], serving as a critical tool in biomedical research. These models have been instrumental not only in advancing our understanding of disease mechanisms and biological processes but also in evaluating the therapeutic potential and safety of new treatments. Despite these advancements, physiological and genetic variations, especially in immune system organization and organ-specific toxicity, limit the translation of animal model findings to humans [2,3]. For instance, the immune system of rodents varies considerably from that of humans, and their accelerated metabolic rates may complicate the accurate prediction of human drug-toxicity outcomes [4]. These interspecies differences may hinder the effective translation of drug toxicity results from animal to human scenarios. These limitations, along with ethical concerns about animal experimentation, highlight the need for alternative approaches that can predict human responses without animal testing [5,6].

To address these challenges, global initiatives are advancing New Approach Methodologies (NAMs) within Next-Generation Risk Assessment (NGRA) frameworks [7]. NAMs integrate advanced in vitro systems, computational modeling, multi-omics data, and predictive machine learning approaches to generate human-relevant evidence for decision-making [8,9,10,11,12,13]. Within this framework, in vitro models are a cornerstone, offering physiologically relevant platforms for studying human biology in controlled environments. Two-dimensional (2D) cell cultures are accessible and cost-effective but do not fully reproduce native tissue architecture or cell–cell and cell–matrix interactions, which can influence cellular morphology, gene expression, and toxicant responses [14,15]. Three-dimensional (3D) systems, including spheroids and co-cultures, provide more tissue-relevant structural and cellular contexts [16,17,18,19]. Among these, organoids derived from primary or stem cells can reproduce important aspects of organ-specific architecture and epithelial function, making them useful controlled models for investigating drug-induced responses [20,21,22,23,24,25,26].

Treatment with chemotherapeutics can often lead to off-target adverse effects [27], such as gastrointestinal (GI) toxicities [28], which may result in discontinuation of treatment. Chemotherapy-induced intestinal mucositis (CIM) is a serious complication in patients undergoing chemotherapy and is marked by inflammation-driven breakdown of the intestinal epithelium. CIM has been linked to off-target GI toxicities including nausea, vomiting, diarrhea, and abdominal pain [29,30,31,32], affecting up to 40% of patients and contributing to decreased quality of life and tolerance to treatment [29,30,33,34]. Despite the high incidence of GI toxicities induced by chemotherapeutics, this area remains underinvestigated compared with extensively studied liver and kidney toxicities. Liver and kidney models are heavily studied because these organs are central to drug metabolism and excretion. Animal models may not fully capture human gastrointestinal responses to chemotherapeutics such as doxorubicin (DOX) [28,35]. Colon organoids, also referred to as colonoids, provide controlled human-derived epithelial systems that retain aspects of intestinal physiology [36]. In this study, human colonoids were compared with mouse colonoids and mouse colon to identify conserved and candidate human colonoid-specific responses to DOX.

We used DOX exposure as an example to demonstrate how species-specific differences between human and mouse models can lead to variations in drug-induced adverse GI toxicities. This was investigated through transcriptomic analysis of three models: mouse colon tissue (mouse in vivo), mouse colonoids (mouse in vitro) and human colonoids (human in vitro) using the parallelogram approach in toxicology. The parallelogram approach is a framework in toxicology used to extrapolate human in vivo responses by comparing data from animal in vivo, animal in vitro, and human in vitro models [37]. Using this approach, we aimed to answer two key research questions: (i) How well do mouse in vitro models mimic the mouse in vivo response? (ii) How closely does the human in vitro model align with the mouse in vivo and in vitro models? Accordingly, we tested two hypotheses: first, that mouse colonoids would retain epithelial transcriptional responses comparable to those observed in mouse colon after DOX exposure; and second, that human colonoids would provide an epithelial response profile that enables the identification of model- and species-dependent differences. Together, this comparative framework was used to identify conserved and model-specific DOX-responsive mechanisms that can guide future validation against human clinical or biopsy-derived intestinal data.

## 2. Results

### 2.1. Transcriptomic Changes Induced in Human and Mouse Models

**Figure 1:**
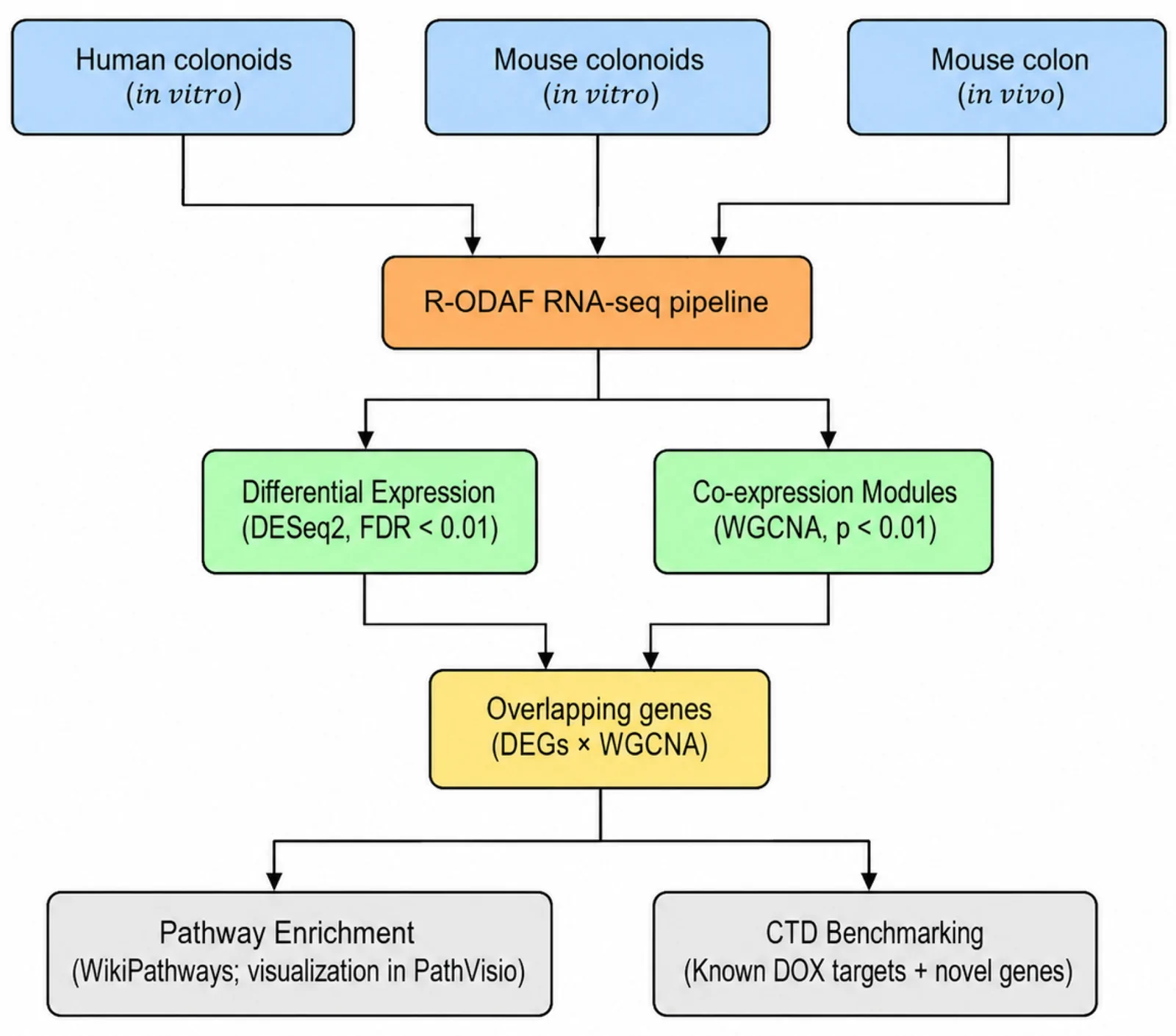
Workflow illustrating the complete transcriptomics analysis pipeline. The schematic shows the study design across human colonoids, mouse colonoids, and mouse colon. RNA-seq data from all models were processed using the R-ODAF pipeline for quality control, trimming, alignment and quantification. DESeq2 differential expression analysis and WGCNA were applied independently to each dataset and significant genes were subjected to pathway analysis and cross-referenced against the CTD.

This study aimed to compare DOX-induced transcriptomic responses across human colonoids, mouse colonoids, and mouse colon within a shared analytical framework. RNA-sequencing (RNA-seq) datasets from all models were processed using the Omics Data Analysis Framework for Regulatory Application (R-ODAF) pipeline. Differential expression analysis and weighted gene co-expression network analysis (WGCNA) were applied independently to each dataset, followed by pathway analysis and cross-referencing against the Comparative Toxicogenomics Database (CTD). Figure 1 provides an overview of the study design and workflow.

**Figure 2:**
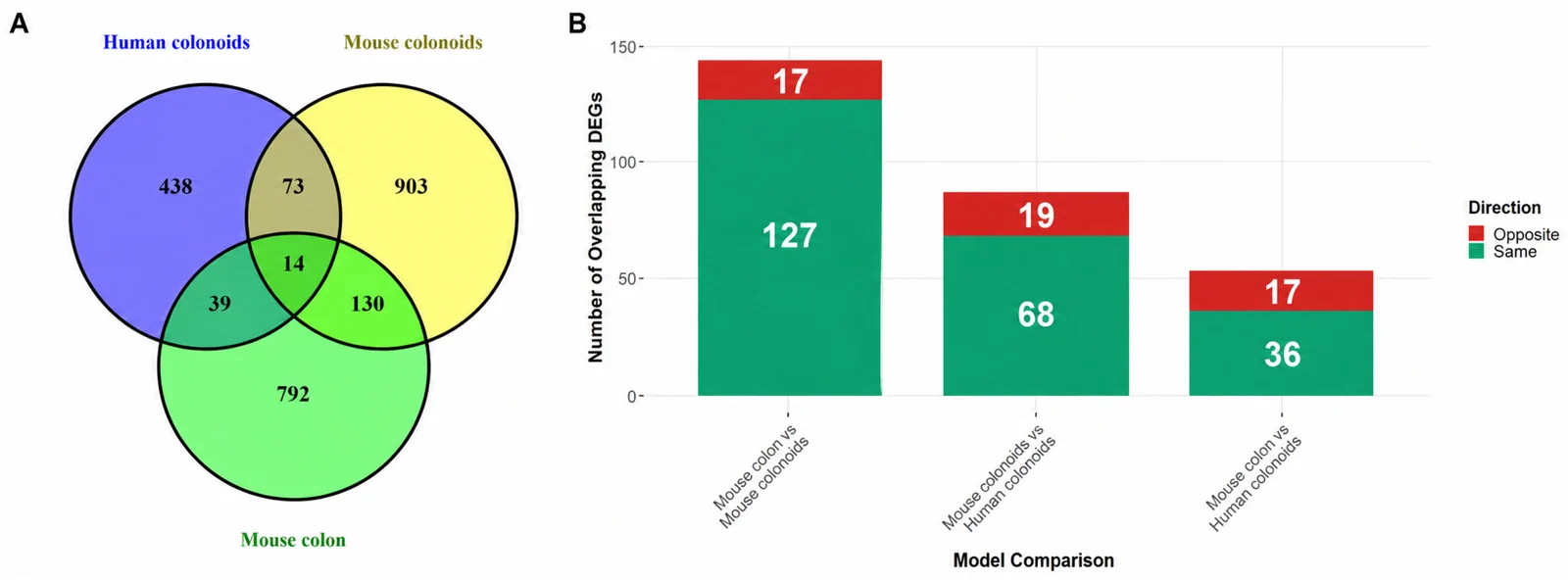
DEG overlap and directional concordance across models. (**A**) Venn diagram representing the number of DEGs unique to or shared between human colonoids, mouse colonoids and mouse colon following treatment with DOX. (**B**) Stacked bar chart showing the number and directionality of overlapping DEGs for each model comparison: mouse colon vs. mouse colonoids, mouse colonoids vs human colonoids, and mouse colon vs human colonoids. “Same direction” (green) indicates that the overlapping DEGs are either upregulated or downregulated in the models compared. “Opposite direction” (red) indicates that the overlapping DEGs are upregulated in one model and downregulated in the other.

To characterize the transcriptional responses to DOX, differential gene expression analysis was performed on human colonoids, mouse colonoids, and mouse colon (Figure 2A) to identify differentially expressed genes (DEGs; false discovery rate [FDR] < 0.01). Prior to differential expression analysis, principal component analysis (PCA) was performed separately for each dataset to evaluate transcriptional structure and sample variability (Figure S1). In all models, the first principal component (PC1) distinguished DOX-treated from control samples, indicating that treatment accounted for the largest source of expression variance, with dose and time contributing secondary sources of within-group variation. Because the exposure designs differed across datasets, DOX-treated samples were aggregated across the available doses and time points within each model to estimate an overall treatment-associated transcriptional response. This approach supported broad cross-model comparison while placing less emphasis on individual dose- and time-specific patterns. A total of 564 DEGs were identified in human colonoids, 1120 in mouse colonoids, and 975 in mouse colon (Figure 2A, File S1). Across all three datasets, 14 DEGs were identified (Figure 2A and Figure S2a), including *BAX*, *INKA2*, *PCNA* and *POLR2H*. The majority of these 14 shared genes showed a similar expression direction except for *PCNA* and *POLR2H*, which were downregulated in mouse colonoids and upregulated in the other two models. A comprehensive overview of the shared DEGs across all three models is provided in Table 1.

To assess concordance at the gene level, expression trends of overlapping DEGs were analyzed across datasets to identify patterns of directional agreement or divergence (Figure 2B and Figure S2b,d). The highest directional concordance was observed between mouse colon and mouse colonoids (88.2%; Figure S2c), suggesting substantial transcriptional similarity between the in vitro and in vivo mouse systems. A lower but still substantial concordance was found between human and mouse colonoids (78.2%, Figure S2b), whereas human colonoids and mouse colon showed the lowest concordance in the gene expression direction (Figure S2d), with just 67.9% of overlapping genes exhibiting the same expression change. Several overlapping DEGs showed inverse regulation between models. For example, *PCNA* and *SLC7A11* were upregulated in human colonoids but downregulated in mouse colonoids. Similarly, *RAD51* and *RRM2* were downregulated in human colonoids and upregulated in mouse colon, underscoring a model-dependent transcriptional response to DOX. Detailed gene-level lists of overlapping DEGs, including directionality across model pairs, are provided in File S1.

In a secondary sensitivity analysis excluding the 6 h mouse colon samples, the number of overlapping DEGs increased from 53 to 80 for human colonoids versus mouse colon and from 144 to 178 for mouse colonoids versus mouse colon. However, directional concordance decreased from 67.9% to 48.8% and from 88.2% to 67.4%, respectively. Thus, the acute 6 h time point was not the sole explanation for the observed cross-model discordance. Detailed results are provided in File S1.

### 2.2. Identification of Significant WGCNA Module Genes

To identify clusters of co-expressed genes responsive to DOX treatment, WGCNA was applied to each dataset to detect modules that were significantly associated with dose and time. WGCNA was performed across all samples within each dataset. Module eigengenes were correlated with overall treatment status, reported as “Dose + Time” in Figure S3, as well as with dose and time separately. Modules significantly associated with the overall treatment-status trait were selected for downstream analysis. Gene significance was determined by the correlation of each gene’s expression profile with the observed traits. In total, three significant modules were identified in human colonoids and five each in mouse colonoids and mouse colon based on *p*-value < 0.01 (Figure S3a–c).

Following module detection, genes from all significant modules in each dataset were aggregated to form a comprehensive gene set for further analysis. A total of 1305 genes from human colonoids, 3108 from mouse colonoids, and 1951 from mouse colon were identified. A complete overview of the number of DEGs and genes per significant module is provided in Table S2. In addition, a pairwise Jaccard overlap analysis was conducted, in which each significant module from one dataset was compared with all modules in the other datasets (Figure S4) to quantify gene-set overlap. The highest similarity appeared within the mouse models, with a peak value of 0.11 between mouse colonoid Module 8 and mouse colon Module 15. By contrast, the overlap between mouse and human colonoids was low (maximum J < 0.05), and the overlap between mouse colon and human colonoids was likewise limited (J = 0.04), indicating few shared genes in the cross-species comparison.

### 2.3. Pathways Identified Through DEG and WGCNA Gene Sets

Pathway analysis was performed on both DEGs and gene sets derived from significant WGCNA modules (File S1), using an adjusted *p*-value < 0.05 to define significance. DEG-based enrichment identified 30 significant pathways in human colonoids, 56 in mouse colonoids, and 19 in mouse colon (Table S3), revealing both shared and model-specific responses. Pathways related to cell-cycle regulation, DNA damage, and DNA repair were enriched in all three models, indicating transcriptomic signatures consistent with the involvement of these processes despite differences among systems. Pathway analysis on gene sets derived from significant modules yielded 10 pathways in human colonoids, 54 in mouse colonoids and 52 in mouse colon. Similarly, processes related to cell cycle, DNA damage response, and DNA repair that were enriched among DEGs were also significant in WGCNA pathways.

Within each dataset, the overlap between DEG- and WGCNA-based enrichment comprised four pathways in human colonoids, 17 in mouse colonoids and eight in mouse colon. Notably, the overlapping pathways in all three models converged on processes involved in DNA damage, including the p53 signaling cascade, miRNA-regulated DNA damage response, and encompassed cell-cycle regulation and DNA-repair mechanisms, illustrated by cell-cycle checkpoint control, cell cycle arrest, and DNA mismatch repair. A concise overview of the major conserved and model-associated pathway themes supported by significant enrichment results is provided in File S1, worksheet “Pathway_Summary.”

### 2.4. Gene-Level Mapping to DOX-Related Pathways

Comparison of enriched pathways across human colonoids, mouse colonoids and mouse colon provided a framework for mapping genes of interest (GOIs), defined within each dataset as genes that were both differentially expressed and members of significant WGCNA modules. These GOIs were mapped to cell-cycle regulation, DNA damage response, DNA repair, and programmed cell death pathways to compare model-level responses to DOX. Most GOIs mapped to these processes were unique to each dataset, indicating model-specific transcriptional changes in response to DOX exposure.

#### 2.4.1. Cell Cycle Arrest and Programmed Cell Death

**Figure 3:**
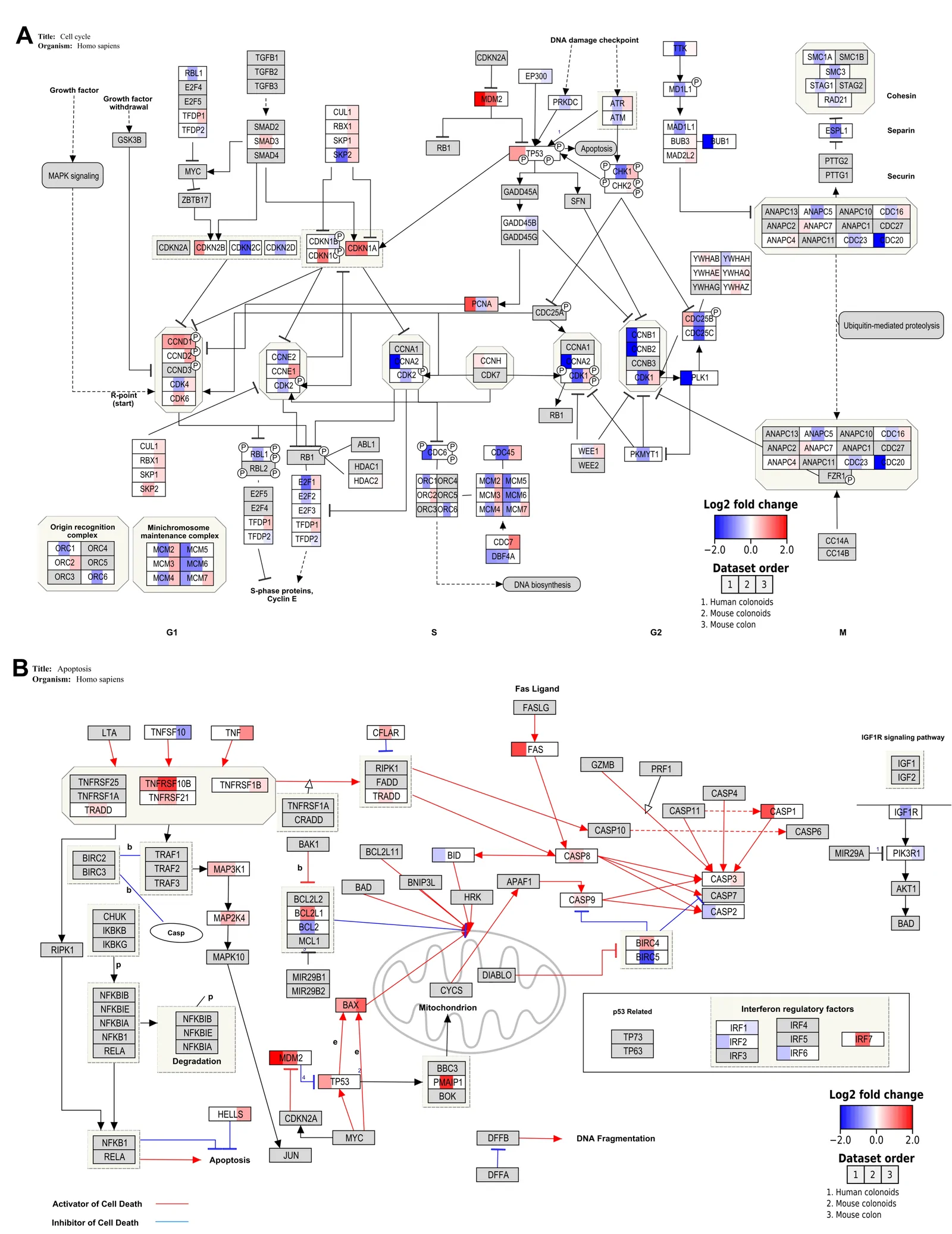
Gene expression mapping on (**A**) cell cycle and (**B**) apoptosis pathway in response to doxorubicin in all three models. Each gene is depicted as a vertically segmented box colored by log_2_(fold-change) (−2 = blue, 0 = white, +2 = red), with the left segment representing human colonoids, the middle segment mouse colonoids, and the right segment mouse colon.

A substantial number of GOIs from each dataset were mapped to the cell-cycle pathway (Figure 3A), with DNA-damage checkpoint components consistently represented across all models. *CDKN1A* (p21), a checkpoint effector, and *MDM2*, the regulator of p53, were present in both human and mouse colonoids and were upregulated in each. *Atr* and *Chk1* were shared by the two mouse datasets but showed opposite gene expression directionality, being upregulated in mouse colon and downregulated in mouse colonoids indicating model-specific regulation of these kinases. The majority of genes common within the mouse systems likewise displayed opposite regulation. For example, *Cdc16* (a subunit of the anaphase-promoting complex) was elevated in mouse colonoids but suppressed in mouse colon, whereas origin-licensing factors (e.g., *Mcm2*, and *Mcm4*) followed an inverse pattern, being upregulated in mouse colon and downregulated in mouse colonoids.

To determine how DOX shapes programmed cell-death pathways in each model, GOIs were also mapped onto the apoptosis pathway (Figure 3B). The pathway mapping revealed that most of the genes mapped exclusively to a single model. *BAX*, a pro-apoptotic BCL-2 family effector, was the only gene common to all three datasets and was uniformly upregulated, consistent with a shared transcriptional signature of intrinsic apoptosis. By contrast, the death receptor gene FAS appeared only in the human colonoids within the present comparison, where it was upregulated, indicating a candidate extrinsic-apoptosis response requiring further validation. *MDM2*, also identified in the cell-cycle pathway, was present in both colonoid models and was upregulated. *TP53*, also mapped to the cell-cycle pathway (Figure 3A), was detected in the apoptosis pathway in human colonoids, reflecting p53’s central role in coordinating cell cycle arrest and apoptotic execution after DOX exposure. *Map2k4* was the only apoptosis-related gene common to the mouse models, and no other apoptosis-related genes overlapped within the mouse systems. By contrast, genes shared between the two colonoid models displayed the same directionality of expression (Figure 3B).

#### 2.4.2. DNA Damage Response and Repair

**Figure 4:**
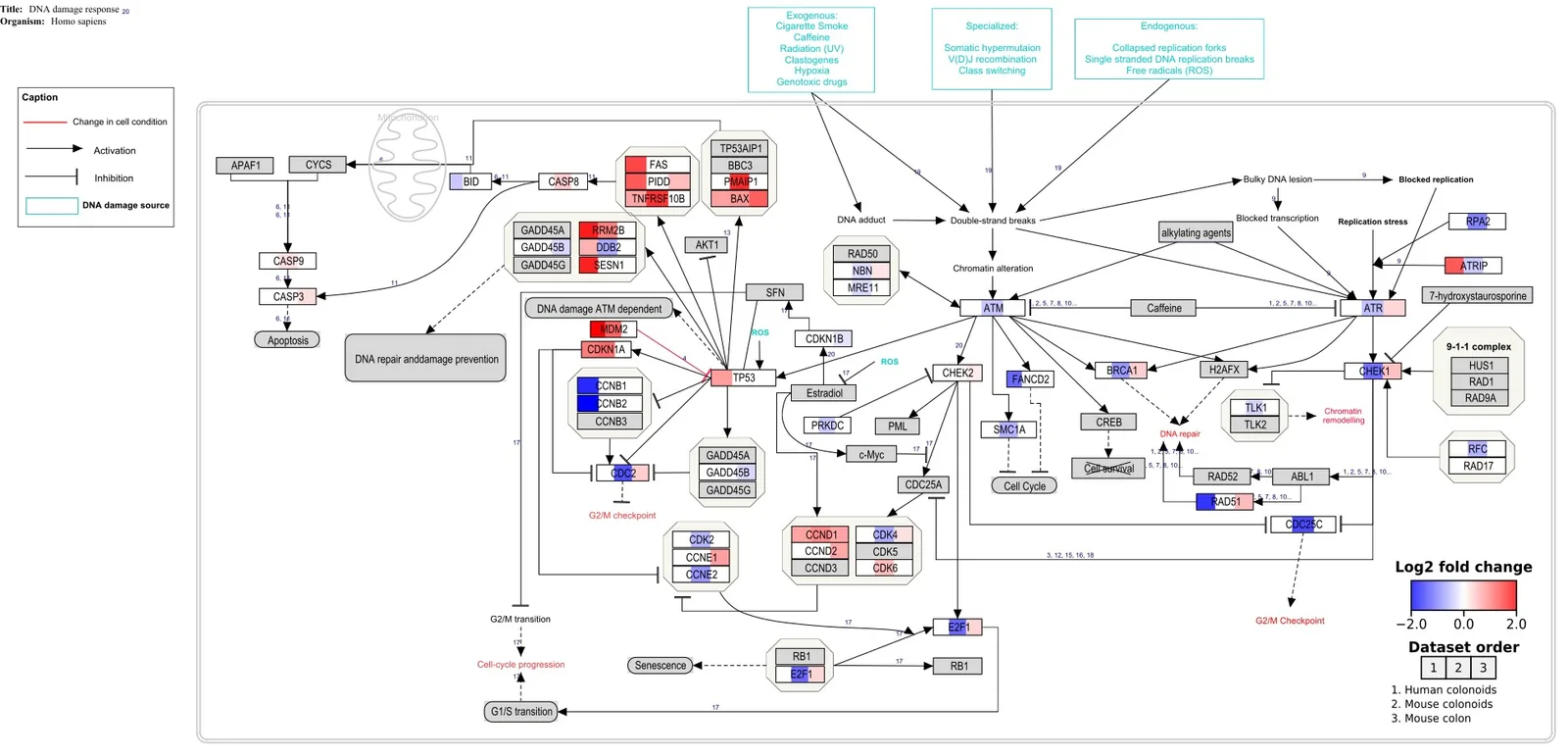
Gene mapping on DNA damage response pathway in response to doxorubicin in all three models. Each gene is depicted as a vertically segmented box colored by log_2_(fold-change) (−2 = blue, 0 = white, +2 = red), with the left segment representing human colonoids, the middle segment mouse colonoids, and the right segment mouse colon.

Overlaying the GOIs on the DNA damage response (DDR) pathway (Figure 4) revealed only two genes, *BAX* and *CCND1*, shared by all three models. The remaining genes were largely model-specific. Among the genes common to both mouse colon and colonoids, expression patterns were consistently opposite. For example, *Brca1* and *Cdk4* were upregulated in mouse colon and downregulated in mouse colonoids, and the checkpoint kinase *Atr* followed the same inverse trend, indicating divergent regulation of DNA repair and checkpoint signaling between the in vivo tissue and its organoid counterpart. In contrast, *RRM2B* and the previously noted cell-cycle regulators *MDM2* and *CDKN1A* (Figure 3A) were all upregulated in both colonoid datasets (Figure 4). Human colonoids also displayed several unique DDR genes, including *FAS* and *SESN1*, which are associated with p53-driven repair and stress-response pathways not identified in the mouse models.

In addition to DDR pathways, the GOIs were mapped to the DNA repair pathways (Figure S5). Mapped DNA-repair genes were both model-specific as well as sporadically overlapping. Genes shared between mouse colon and mouse colonoids displayed opposite gene expression except for a few genes such as *Mgmt* and *Polk*, showing upregulation across the two systems. Similarly, genes common between human and mouse colonoids showed concordant regulation, consistent with the patterns observed in earlier pathways.

### 2.5. CTD Contextualization of Model-Derived Genes

**Figure 5:**
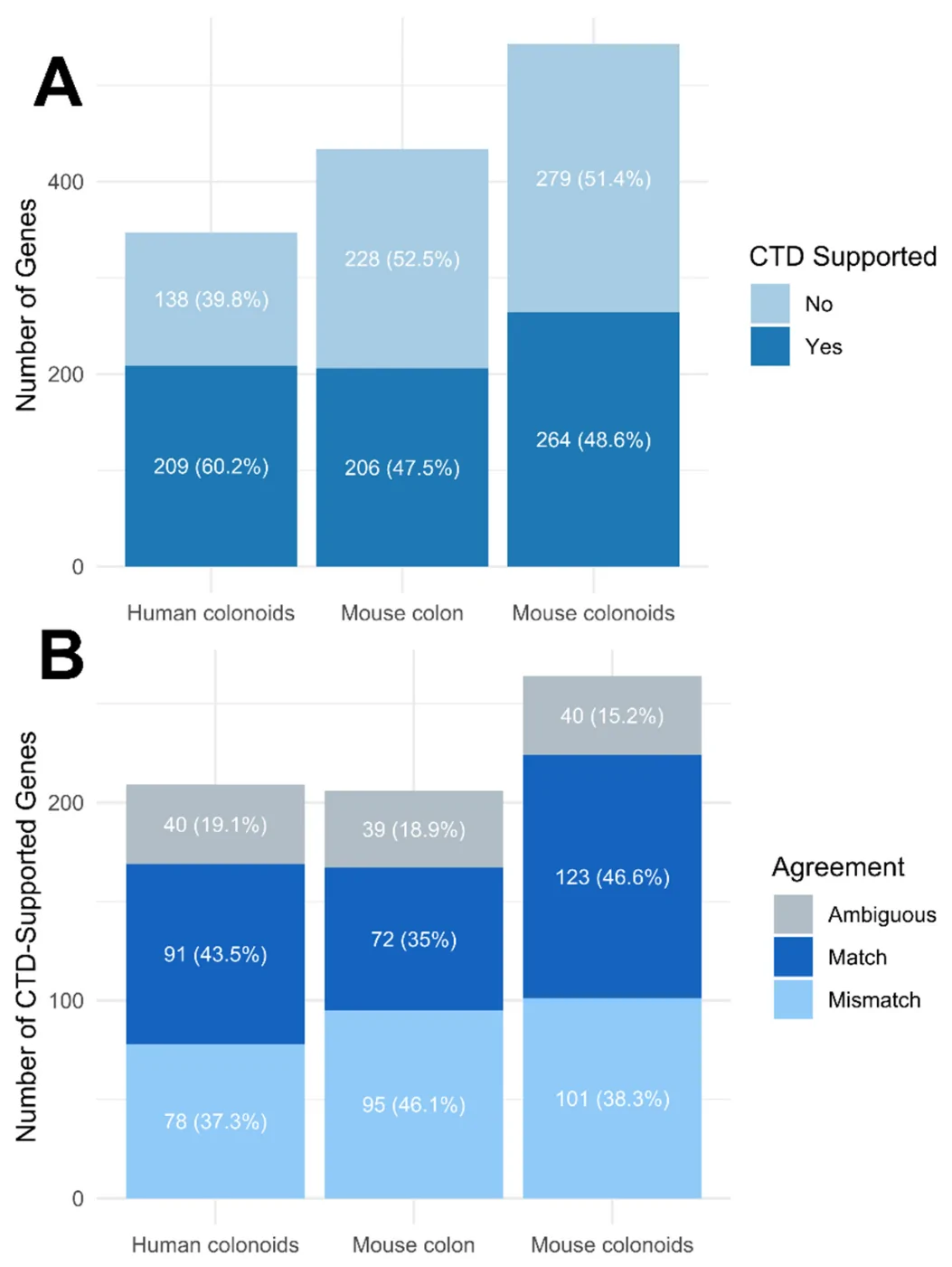
Cross-referencing the overlapping DEGs and WGCNA genes against CTD. (**A**) CTD annotation coverage of doxorubicin-responsive genes across models. (**B**) Displays the agreement based on match, mismatch, or ambiguous among the CTD-supported genes (209 in human colonoids, 206 mouse colon, and 264 mouse colonoids).

To further contextualize the model-derived signatures, overlapping genes from the DEG and WGCNA analyses were queried in the CTD, linking the transcriptomic findings to curated chemical–gene interaction data (File S1) and allowing assessment of their prior documentation in the context of DOX exposure. The resulting comparison showed that cross-referencing of the overlapping DEGs and WGCNA genes against the CTD revealed that 60.2% of human colonoid genes had prior CTD annotations for DOX, in contrast to 48.6% in mouse colonoids and 47.5% in mouse colon (Figure 5A). Both mouse models displayed a similar number of DOX-interacting genes compared to human colonoids, yet their larger gene sets lowered their relative CTD coverage. Notably, a substantial proportion of model-derived genes, totaling 619 unique genes identified through an SQL query (645 when summed across models in Figure 5A), were not present in the CTD interaction dataset used (File S1), which may reflect underreported or context-specific DOX-response signals, as well as differences in the available CTD evidence. Figure S6a–c highlights the top five upregulated and downregulated genes lacking CTD annotation in each dataset. In human colonoids, the most upregulated genes included *EFCAB10* and *HRNR* and the most downregulated genes included *PADI1* and *KIF24*. In mouse colonoids, *Cox6b2* and *Dsg3* showed the greatest upregulation and *Ooep* and *Retnlb* were the most downregulated. In mouse colon, *Ddit4l* and *Mmp7* were the most upregulated genes, whereas *Wdfy4* and *Hla-Dob* were the most downregulated genes.

#### Directionality Concordance with the CTD

To further benchmark model-derived transcriptional changes against the literature, CTD’s curated “increase/decrease” annotations for doxorubicin–gene interactions were used as reference. Comparing the CTD’s directional annotations with the GOIs indicated 43.5% concordance in human colonoids, 46.6% in mouse colonoids, and 35% in mouse colon, revealing distinct levels of concordance between human and mouse models (Figure 5B). Here “Match” denotes genes whose CTD-reported effect agrees with the observed up/downregulation, “Mismatch” represents the genes whose CTD annotation is opposite to our observed gene expression, and “Ambiguous” denotes genes for which CTD’s interaction terms do not clearly specify directionality. The proportion of mismatched genes was highest in mouse colon (46.1%) and lowest in human colonoids (37.3%). Ambiguous CTD annotations made up approximately 15 to 20% of the overlapping genes list across the three datasets. This comparison illustrates how model-specific expression aligns with curated knowledge, identifying both conserved responses and model-dependent variations. Both CTD coverage and directional concordance were interpreted descriptively and were not used to infer statistically significant differences between models.

## 3. Discussion

In this study, human colonoids were evaluated as a potential human-relevant, epithelium-focused in vitro model for GI toxicity using DOX, and transcriptomic responses were compared across human colonoids, mouse colonoids, and mouse colon in a parallelogram framework. GI adverse effects are common among chemotherapy recipients and can limit therapeutic dosing and overall treatment success [33]. Because exposure designs were not fully matched (mouse colon included a 6 h time point and two bolus doses, whereas colonoids were first sampled at 24 h under continuous exposure), analyses were pooled across time and dose and cross-model differences should be interpreted accordingly. This pooling strategy prioritized robust DOX-associated transcriptional signatures for cross-model comparison rather than dose- or time-specific dynamics.

Mouse colon profiles were generated from mucosal scrapings, whereas colonoids represent an epithelial-focused compartment, providing complementary tissue- and epithelium-resolved views of the DOX response and likely contributing to some of the observed cross-model differences. The sensitivity analysis excluding the 6 h mouse colon samples increased DEG overlap but reduced directional concordance from 67.9% to 48.8% for human colonoids versus mouse colon and from 88.2% to 67.4% for mouse colonoids versus mouse colon (File S1). This indicates that the acute sampling time was not the sole explanation for the observed differences, which likely also reflect the exposure regimen, tissue composition, and model-specific transcriptional dynamics. The pooled analysis was therefore retained as the primary analysis.

Differential expression and co-expression network analyses captured both gene-level and coordinated responses to DOX, including conserved cytotoxic programs shared across datasets and model- or species-specific signatures. The strongest DEG overlap was observed between mouse colon and mouse colonoids, with high directional concordance (88.2%; Figure S2c), suggesting that mouse colonoids capture components of the DOX-responsive transcriptional program observed in mouse colon. By contrast, the lowest concordance was observed between the shared DEGs between human colonoids and mouse colon at 67.9% (Figure S2d). This lower concordance may partly reflect differences in cellular composition, because mouse colon mucosal scrapings can include immune, stromal, and other lamina propria-associated signals that are absent from epithelial-focused colonoids. Thus, genes or pathways detected only in mouse colon, or showing different regulation relative to colonoids, may reflect model composition as well as species differences.

Several genes displayed inverse regulation within the mouse systems, including *Psat1* and *Hspb1* (File S1). *Hspb1* encodes a small heat-shock protein whose phosphorylation regulates cytoskeleton recruitment and cell motility [38]. Its upregulation in mouse colon and downregulation in mouse colonoids suggests a protective chaperone response to DOX-induced stress that may be less represented in the organoid model.

Comparison of human and mouse colonoids revealed several shared genes with inverse regulation, including *PCNA* and *SLC7A11* (File S1). *SLC7A11*, the cystine/glutamate antiporter that supports glutathione synthesis, was downregulated in human colonoids but upregulated in mouse colonoids, suggesting greater susceptibility to DOX-induced oxidative stress in human colonoids and a potential stress-adaptive response in mouse colonoids. Inverse regulation was also observed between human colonoids and mouse colon, including *RRM2* and *RAD51*. *RRM2* encodes a subunit of ribonucleotide reductase and was strongly downregulated in human colonoids but only modestly increased in mouse colon, consistent with reduced DNA synthesis capacity and greater DOX sensitivity in the human model. *RRM2* is frequently overexpressed in cancer cells, and its inhibition has been reported to enhance anticancer activity [39]. These patterns can alter how DOX-triggered pathways unfold in each system and highlight the importance of which genes are engaged, as well as their direction and magnitude of expression.

Differential gene expression analysis identified 14 genes shared across human colonoids, mouse colonoids, and mouse colon, representing a minimal conserved DOX response enriched for p53-linked DNA damage and apoptosis programs (Table 1, Figure 2A and Figure S2a). *BAX*, a pro-apoptotic effector, was upregulated in all three models, consistent with p53-dependent intrinsic apoptosis activation [40]. *INKA2*, a direct p53 target [41], was also consistently upregulated, supporting conserved growth inhibition following DNA damage [42]. In contrast, *PCNA* and *POLR2H* were downregulated in mouse colonoids but upregulated in human colonoids and mouse colon, with *PCNA* showing the strongest increase in human colonoids. WGCNA identified DOX-responsive modules, with greater overlap between mouse colon and mouse colonoids than between mouse and human colonoids. Pairwise Jaccard indices (Figure S4) showed low overlap in exact WGCNA module gene membership, with no comparison exceeding 0.11. However, because modules were generated independently in each dataset, different genes or pathway components could still converge on the same p53-associated, cell-cycle, DNA damage response, DNA-repair, and apoptosis processes.

Enrichment of DEGs and significant WGCNA modules converged on cell-cycle checkpoints, p53-mediated apoptosis, and DNA repair, highlighting conserved DOX toxicity biology. GOIs were mapped to cell-cycle, apoptosis, DNA damage response and DNA repair pathways (Figure 3A,B, Figure 4, and Figure S5) to compare model- and species-specific pathway-level gene shifts.

Pathway mapping identified transcriptomic signatures consistent with cell-cycle checkpoint engagement across models (Figure 3A). In both human and mouse colonoids, the p53 targets *CDKN1A* and *MDM2* were upregulated, and *TP53* was elevated in human colonoids, consistent with p53-dependent G1/S control and pathway engagement [43,44,45]. In contrast, the checkpoint kinases *Atr* and *Chk1* showed upregulation in mouse colon and downregulation in mouse colonoids, suggesting model-specific replication stress handling [46]. Replication-licensing factors (*Mcm2*, *Mcm4*) followed the same pattern, with upregulation in mouse colon and downregulation in mouse colonoids. Elevated *Mcm2* has been linked to increased DOX sensitivity in cancer cells [47], consistent with a more proliferative and potentially DOX-vulnerable state in mouse colon versus cell-cycle exit and reduced sensitivity in mouse colonoids.

Apoptosis pathway mapping indicated DOX-induced programmed cell death across models with species- and model-specific features (Figure 3B). *BAX*, a core effector of intrinsic apoptosis [40,48], was upregulated in all three systems and its consistent induction supports a p53-linked intrinsic apoptotic response to DOX in both species [45]. Beyond this core, apoptosis programs diverged. *FAS* was detected only in human colonoids and was upregulated, consistent with death receptor (extrinsic) signaling. *MDM2* was upregulated in both colonoid models, whereas *TP53* mapped to apoptosis only in human colonoids, suggesting stronger p53 coupling to apoptotic execution in the human system. *Map2k4* was the only apoptosis gene shared between mouse colon and mouse colonoids, implicating stress-activated Mkk4–Jnk/p38 signaling in murine responses. Directionality among apoptosis genes shared between human and mouse colonoids was concordant.

Analysis of the DDR pathway showed distinct regulatory signatures (Figure 4). In human colonoids, downregulation of *CCNB1* and *CCNB2* indicated G_2_/M checkpoint arrest, while upregulation of *FAS*, *TNFRSF10B*, and *PID1* supported engagement of extrinsic apoptosis. Both colonoid models mapped *MDM2* and *CDKN1A* to p53 pathway activation, but only human colonoids engaged *RRM2B*, *DDB2*, and *SESN1* together, consistent with a broader p53-dependent repair program. Mouse colonoids showed *Rrm2b* upregulation with *Ddb2* downregulation, whereas mouse colon did not display a strong repair signature. Homologous recombination factors also differed, with *RAD51* downregulated in human colonoids and *Brca1* downregulated in mouse colonoids, while both were upregulated in mouse colon. *BAX* was also upregulated in DDR across all models (Figure 4), supporting conserved intrinsic apoptosis engagement. *TP53* and *CASP1* were elevated only in human colonoids, whereas *Map3k1* and *Map2k4* were upregulated only in the mouse datasets, indicating candidate model- and species-associated stress-signaling routes. Overall, GOIs showed the most consistent overlap and directionality between human and mouse colonoids, with lower overlap and greater divergence versus mouse colon.

RNA-seq provided a common molecular endpoint across all three systems and enabled direct cross-model comparison. The identified p53, apoptosis, and DNA-damage pathways should therefore be interpreted as coordinated transcriptomic signatures consistent with the involvement of these processes rather than as direct functional confirmation. A matched phenotypic, histological, or protein-level validation dataset was not available across all three systems. Future studies integrating matched functional, histological, and protein-level endpoints across models will further strengthen the mechanistic interpretation of these transcriptomic findings.

To contextualize the model-derived GOIs, they were cross-referenced against the CTD for DOX interactions. Human colonoids showed the highest coverage, with 60.2% of GOIs annotated for DOX, compared with 48.6% in mouse colonoids and 47.5% in mouse colon (Figure 5A). The mouse models had similar absolute counts of the CTD-listed genes, yet their larger GOI counts lowered the proportional coverage. These differences may reflect variation in the literature coverage, experimental context, or biological response and cannot be attributed to a single factor.

Several DOX-responsive genes were not listed in the CTD yet have plausible roles in gastrointestinal physiology and barrier function. In human colonoids, *HRNR* and *PADI1* were among the most dysregulated genes (Figure S6a). *HRNR*, a cornified envelope-associated protein with antimicrobial domains [49], was strongly upregulated, identifying it as a candidate for further investigation in epithelial stress and barrier-related responses. Whether HRNR induction is associated with altered epithelial barrier properties, such as permeability or remodeling, requires targeted functional validation.

*PADI1* encodes peptidyl arginine deiminase 1, which mediates protein citrullination, and was the most downregulated gene in human colonoids. *KIF24* encodes a centriole-associated kinesin reported to regulate ciliogenesis via *CP110* and *CEP97* [50] and was also downregulated, identifying ciliogenesis-related processes as a hypothesis for future investigation. In mouse colonoids, *Dsg3*, a desmosomal cadherin essential for cell–cell adhesion [51], was among the most upregulated genes (Figure S6b), whereas *Retnlb* (RELMβ), implicated in mucosal barrier function and intestinal inflammation [52], and *Asrgl1* were among the most downregulated. In mouse colon, *Ddit4l* and *Mmp7* were strongly upregulated (Figure S6c). *Mmp7* has been linked to barrier dysfunction through junctional disruption, including claudin-7 degradation [53]. Collectively, these CTD-missing yet GI-relevant signals highlight coverage gaps in GI-focused toxicogenomics. Concordance variations in Figure 5B underscore how different models capture distinct biological signatures while revealing gaps in current toxicogenomic annotation. Together, these findings support the value of cross-model evidence for improving intestinal toxicity annotation and prioritizing candidates for future functional validation. These gene-level interpretations are hypothesis-generating, and functional measurements would be required to determine whether the observed transcriptional changes are accompanied by altered barrier function, protein citrullination, junctional organization, or ciliary biology.

Despite species-level differences and the absence of stromal or systemic context in organoids, each model captured a common core of CTD-documented DOX targets. Although gene sets differed between models, the DOX response remained centered on the same core pathways, indicating conserved mechanisms despite system-specific gene differences. Directionality analysis comparing CTD-reported increases/decreases with observed GOI up- or downregulation showed modest concordance (46.6% in mouse colonoids, 43.5% in human colonoids, and 35% in mouse colon; Figure 5B). These values indicate partial agreement with CTD annotations and should be interpreted as contextualization rather than external validation. The mouse in vivo model showed the highest mismatch fraction, while ambiguous CTD direction annotations (no clear direction) comprised ~15–20% of overlapping genes across systems. This modest concordance may reflect differences in tissue and cellular composition, species, exposure conditions, and the heterogeneous experimental evidence represented in the CTD. Therefore, CTD-missing or directionally discordant genes should not be considered evidence of biological novelty alone. Consistent with Figure S6, several DOX-responsive GOIs lacked CTD entries but had plausible GI-relevant functions, supporting their prioritization for functional follow-up. Conserved p53/DDR and checkpoint programs in human colonoids suggest candidate epithelial mechanisms that can be tested in DOX-treated patients or clinical intestinal samples. These findings warrant benchmarking against intestinal biopsy transcriptomics and stool-based epithelial injury markers.

The higher DEG-level directional agreement between mouse colonoids and mouse colon, 88.2% (Figure S2c), suggests that mouse colonoids capture part of the in vivo mucosal response to DOX. However, pathway-level mapping of genes involved in cell-cycle regulation, apoptosis, and DNA damage response (Figure 3A,B and Figure 4) indicates that this agreement was not uniform across all mapped genes, with some responses appearing more similar between the two colonoid systems. These findings partially support the first hypothesis, as mouse colonoids reflected part, but not all, of the mouse in vivo transcriptional response. The pathway-level mapping results add an important nuance, indicating that some DOX responses may be shared by the colonoid context rather than species alone. The second hypothesis was also supported within the scope of this study, as human colonoids provided an epithelial response profile that enabled the identification of candidate model- and species-associated differences. However, the human in vivo relevance of these differences remains to be validated. Human colonoids should therefore be interpreted as controlled human epithelial models for identifying candidate toxicity mechanisms rather than as demonstrably more predictive than mouse models. Their in vivo relevance and relative predictive performance require validation against DOX-specific clinical or biopsy-derived intestinal data. The parallelogram framework conceptually links animal in vivo, animal in vitro, human in vitro, and human in vivo responses. In the present study, the first three were directly evaluated, whereas the human in vivo response remained the target of extrapolation [37]. The absence of comparable DOX-specific human intestinal in vivo data limits validation of this extrapolation and these conclusions regarding clinical predictivity.

Clinical benchmarking of intestinal organoids has been explored in a capecitabine/5-FU study, where healthy colon biopsies from treated patients were compared with 5-FU-exposed human colon organoids [54]. The study identified chemotherapy-associated changes in transport, cellular stress, folate metabolism, and immune-related pathways in patient colon tissue, with selected molecular responses also reflected in the human colonoid model. In the context of the present study, such human-to-human reference data would also help interpret the cross-species comparison by clarifying the extent to which mouse colonoids and mouse colon reflect human intestinal toxicity biology. However, the human colonoid dataset was derived from a single organoid line established from healthy colonic tissue from a 67-year-old male donor [26]. Although replicate cultures enabled characterization of the DOX response within this model, they did not capture inter-individual variability.

Taken together, CTD benchmarking and GOI-based pathway mapping prioritize candidate genes within coordinated cell-cycle, DDR, and apoptosis programs, including CTD-missing yet GI-relevant targets, for functional follow-up of checkpoint control, repair fidelity, and barrier integrity. However, colonoids lack a vascular system for nutrient and oxygen exchange and do not reproduce systemic processes such as bile acid recycling between the intestine and liver [55,56], which may be essential for fully understanding systemic chemotherapy responses.

An additional limitation is that both murine datasets were generated exclusively from male C57BL/6J mice, which limits the generalizability of the findings across sexes. Sex-associated differences have been reported in doxorubicin pharmacokinetics and in experimental outcomes including tissue injury, DNA damage, apoptosis, and transcriptional responses [57,58,59]. However, most available evidence comes from cardiac toxicity and systemic studies, so it remains uncertain whether comparable sex-dependent effects occur in the intestine. The present findings therefore apply to the male systems examined, and the identified p53-associated DNA-damage, cell-cycle, and apoptotic responses should not be assumed to be sex-independent. Nevertheless, these findings provide a reference for future studies of sex-specific differences.

Integrating human colonoids with complementary systems that add missing biology, including epithelial–immune or epithelial–stromal co-cultures and intestinal microphysiological platforms, together with refined intestinal toxicity datasets, can provide a more human-centric framework to assess DOX and other chemotherapeutics [28], including 5-fluorouracil, irinotecan, and cisplatin, with minimal reliance on animals. In line with the TransQST perspective on quantitative systems toxicology-enabled drug safety prediction [13], our cross-model transcriptomic comparisons provide transcriptomic evidence and candidate biomarkers that can support the development and validation of GI-focused NAM workflows. Furthermore, human in vivo transcriptomic data will be essential to validate and calibrate human colonoid responses, with particular attention to differences in cellular composition between mucosal scrapings and epithelial-only cultures, ensuring that in vitro-to-in vivo inferences reflect patient outcomes.

## 4. Materials and Methods

### 4.1. Study Design and Model Systems

This study applied a comparative transcriptomic framework to investigate DOX-induced GI toxicity across in vitro human colonoids, in vitro mouse colonoids, and in vivo mouse colon. All datasets were processed through a common RNA-seq workflow [60] to reduce bias and ensure comparability across species. The analytical steps are summarized in Figure 1. The RNA-seq datasets generated and/or analyzed in this study are available in BioStudies under accession numbers E-MTAB-16537 and E-MTAB-16565. The code used for this analysis is available at https://github.com/saadlodhi0916/DOX-GI-Transcriptomics (accessed on 2 August 2026).

### 4.2. In Vivo Model

#### Mouse Colon

All procedures were conducted in an Association for Assessment and Accreditation of Laboratory Animal Care (AAALAC)-approved facility in accordance with relevant animal-welfare legislation and approved by the institutional ethics committee. The in vivo mouse doxorubicin study design and animal procedures were performed as previously described [61]. Briefly, male C57BL/6J mice (10 weeks old) received doxorubicin (5 or 10 mg/kg) by intravenous bolus injection on days 0 and 1, and were euthanized at 6, 24, 72, or 96 h (Table S1) after treatment initiation (with the 6 h group sampled prior to the second dose). Vehicle controls were dosed on the same schedule as described in the original study. Colon mucosal samples were collected for transcriptomic profiling following the reported procedures, with related tissue collection and sample handling procedures described previously [62]. For the in vivo component, the experimental unit was the individual mouse.

### 4.3. In Vitro Models

#### 4.3.1. Mouse Colonoids

Murine colonoids were established using intestinal organoid culture principles adapted from a previous study [63]. Briefly, colons from freshly excised adult male C57BL/6J mice were opened longitudinally, and thoroughly rinsed with ice-cold phosphate-buffered saline (PBS). Crypts were isolated using EDTA-based chelation (5 mM EDTA in PBS, 2 h at 4 °C with gentle agitation) followed by vigorous shaking in sucrose–sorbitol shaking buffer (43.4 mM sucrose and 59.4 mM sorbitol in PBS) for 2 min, then passed through a 100 µm strainer and collected by centrifugation (200× *g*, 10 min, 4 °C). The pellet was resuspended in growth factor-reduced Matrigel (Corning, Corning, NY, USA, #356237) and plated as 50 µL domes in pre-warmed 24-well plates. Following polymerization (37 °C, 30 min), each dome was overlaid with 500 µL 50% conditioned media from L-WRN cells (ATCC:CRL-3276) [64] and 50% IntestiCult^TM^ Organoid Growth Medium (STEMCELL Technologies, Vancouver, BC, Canada, #06005) and maintained at 37 °C in a humidified incubator (95% air/5% CO_2_). Medium was replaced every 3–4 days, and cultures were passaged every 7–10 days by mechanical disruption in ice-cold PBS using a 27G needle, followed by gentle centrifugation (200× *g*, 10 min, 4 °C) and re-embedding in fresh Matrigel.

Following a 72 h recovery after passaging and transfer to 100% Intesticult, colonoids were exposed to DOX (CAS No. 25316-40-9; Sigma-Aldrich, St. Louis, MO, USA, cat. No. D1515), which was prepared from a dimethyl sulfoxide (DMSO) stock and diluted in culture medium to final concentrations of 0.1, 1, and 10 µM, while vehicle controls received 0.1% DMSO. Treatments were applied for 24, 48, or 72 h, with three independent biological replicates for each concentration and three vehicle controls per time point (Table S1). At the end of each exposure, Matrigel domes were dissolved on ice with cold PBS, and the organoids were pelleted by centrifugation (200× *g*, 5–10 min, 4 °C). The resulting pellets were immediately transferred into RNAlater^TM^ (Thermo Fisher Scientific, Waltham, MA, USA), held at 4 °C overnight, and then stored at −80 °C until RNA extraction.

#### 4.3.2. Human Colonoids

Human colonoid transcriptomic data were obtained from a previously published study [26]. The colonoids were derived from healthy colonic tissue from a single 67-year-old male donor. Human colonoids were exposed to DOX at 1 and 10 µM for 24, 48, or 72 h, with three replicate cultures for each treatment and time point, alongside three untreated controls (Table S1). All replicate cultures originated from the same donor-derived organoid line and therefore represented experimental replication within this organoid model rather than replication across independent donors.

### 4.4. PBPK-Informed Dose Selection for Human and Mouse In Vitro Systems

Nominal doxorubicin concentrations were selected to cover clinically relevant intestinal and systemic exposure ranges informed by physiologically based pharmacokinetic (PBPK) simulations. Human colonoids were exposed to 1 and 10 µM doxorubicin [26]. Mouse colonoids were exposed to 0.1, 1, and 10 µM doxorubicin to span the PBPK-informed exposure range reported for the mouse system [61].

### 4.5. Transcriptomics Profiling

#### 4.5.1. Mouse Samples

Total RNA was extracted from mouse colon tissue (in vivo) and mouse colonoids (in vitro) using the miRNeasy Mini Kit (Qiagen, Venlo, The Netherlands), according to the manufacturer’s protocol. RNA concentration and purity were assessed using a NanoDrop ND-1000 spectrophotometer (260/230 nm and 260/280 nm) and RNA integrity was evaluated using an Agilent 2100 Bioanalyzer (RNA Nano Chips). Samples with RNA integrity number (RIN) > 6 and total RNA yield of at least 200 ng were selected for sequencing. Libraries were prepared using the Lexogen SENSE mRNA-seq kit and sequenced on Illumina HiSeq 2500 (Illumina, San Diego, CA, USA) using 100 bp paired-end reads.

#### 4.5.2. Human Samples

For human colonoids (in vitro), transcriptomic data were obtained from a previously published study [26]. Briefly, organoids were collected in QIAzol Lysis reagent, and RNA was isolated using the miRNeasy Mini Kit (Qiagen). Sequencing libraries were prepared using the Lexogen SENSE mRNA Library Preparation Kit (Lexogen, Vienna, Austria). The purified samples were sequenced using the Illumina NovaSeq 6000 system (Illumina, San Diego, CA, USA).

### 4.6. Data Preprocessing

Following sequencing, data from human colonoid, mouse colonoid, and mouse colon were processed using the R-ODAF pipeline [60]. To ensure cross-dataset consistency and minimize bias, the human colonoid data were reprocessed using the same pipeline as the mouse datasets. FastQC v0.11.5 was used to evaluate sequence quality and only the samples that met the established thresholds were retained. Raw reads were then trimmed using fastp v0.19.8 [65], and aligned to reference genomes (GRCm39 for mouse; GRCh38 for human) with STAR v2.7.9a [66]. Transcript quantification was performed using RSEM (1.3.3) [67] to obtain raw gene expression counts for downstream normalization and differential expression analysis. Within-model analyses were performed independently for each dataset. For subsequent cross-model gene-level comparisons, human Ensembl identifiers were annotated with human gene symbols, whereas mouse Ensembl identifiers were mapped to the corresponding human ortholog gene symbols using BioMart [68]. The resulting mapped gene symbols were used for cross-model comparisons, and the complete cross-species mapping table is provided in File S1.

### 4.7. Identification of Differentially Expressed Genes

Following transcript quantification, differential gene expression analysis was conducted using DESeq2 (1.40.2) [69]. R-ODAF filters were applied (≥75% of samples at ≥1 count per million [CPM]; third-quartile low abundance removal; spike removal), and counts were normalized before testing. Genes altered in response to DOX were identified across the three datasets. Within each model, DOX-treated samples across the available doses and time points were pooled and compared with controls to estimate an overall treatment-associated transcriptional response. This approach was selected to support broad cross-model comparison while dose- and time-specific response patterns were outside the primary scope of the analysis. As a secondary sensitivity analysis, the mouse colon differential expression analysis was repeated after excluding the 6 h samples, using the same workflow and FDR threshold. The resulting DEG set was compared with the unchanged human and mouse colonoid DEG sets to recalculate overlap and directional concordance. However, the pooled analysis including all available time points remained the primary analysis. The FDR threshold of 0.01 was predefined in the standardized R-ODAF workflow [60] and applied consistently across all three datasets. This stringent criterion was retained to limit false-positive findings and ensure consistent cross-model comparison.

### 4.8. Weighted Gene Co-Expression Network Analysis

To identify clusters of highly correlated genes within each dataset, weighted gene co-expression network analysis (WGCNA) was performed using the WGCNA package in R (v1.73) [70]. For each dataset, WGCNA identifies modules, which are groups of genes with coordinated expression patterns that are significantly associated with external traits. Normalized count data served as the input, and the default parameters were applied. Briefly, the goodSampleGenes function was used to identify and remove any outlier genes and the pickSoftThreshold function was used to select the soft-thresholding power for constructing a scale-free network prior to transformation into a topological overlap matrix. WGCNA’s default color module labels were converted to numeric identifiers (e.g., Module 1, Module 2) for consistency across datasets. Each resulting module was assigned a significance score based on the module–trait relationship. Modules with *p* < 0.01 were considered significant. Overall treatment status reported as “Dose + Time” in Figure S3, together with dose and time as separate traits, was included in the module–trait correlation analysis. Modules significantly associated with overall treatment status were selected for downstream analysis. To quantify the overlap between module gene sets across datasets, a pairwise Jaccard index [71] was calculated between each module pair. For two sets A and B, the Jaccard is defined as the size of the intersection divided by the size of the union (|A ∩ B|/|A ∪ B|). A value of 0 indicates no shared genes, 0.1 indicates 10% overlap, and 1 indicates identical gene membership.

### 4.9. Pathway Enrichment and Mapping of DEGs and WGCNA Genes

Pathway enrichment analysis was performed to explore biological responses to DOX and assess mechanistic overlap across datasets. This analysis was conducted independently on DEGs and on genes from the significant WGCNA modules using Enrichr [72], querying WikiPathways for pathway annotations. WikiPathways was selected to provide a consistent pathway-enrichment framework across all datasets and to support direct visualization of model-specific gene-expression changes in PathVisio. Using a single pathway resource also reduced redundancy between overlapping pathway databases and facilitated direct cross-model comparison of enriched processes and their contributing genes. Pathways with adjusted *p*-value < 0.05 were considered significant. To identify biologically relevant signals, functionally similar pathways enriched in both DEGs and WGCNA genes were first identified. These pathways were then compared across models based on shared underlying mechanisms, focusing on functional similarity rather than relying on identical pathway names or database-specific terms. Pathways were selected for detailed visualization when they were supported by both DEG- and WGCNA-based enrichment and represented recurring biological processes across the models, enabling cross-model comparison of their contributing genes. Selected pathways were visualized in PathVisio 3.3.0 [73] by overlaying DEGs and significant WGCNA genes to highlight gene-level contributions and facilitate cross-model comparisons.

### 4.10. CTD-Informed Annotation of DEGs–WGCNA Overlap

To contextualize model-derived gene signatures against existing toxicogenomic evidence, a targeted cross-reference analysis was performed using the CTD [74] to annotate genes with known chemical–gene associations. This cross-reference was implemented in R using an SQLite database and an SQL query to obtain de-duplicated gene-level summaries. All known DOX-interacting genes were retrieved from the CTD (accessed 3 July 2025; File S1) and compared with the intersecting DEG and WGCNA gene sets across all three datasets to assess CTD annotation coverage and directional concordance. CTD coverage was calculated as the proportion of intersecting DEG and WGCNA genes with at least one DOX-related CTD annotation.

Subsequently, the directionality of each CTD-annotated interaction (“increased” or “decreased” expression) was compared with the observed gene-expression changes in the datasets to determine concordance, mismatch, or ambiguity across models. CTD coverage and directional concordance were treated as descriptive measures of agreement with curated doxorubicin–gene associations and were not used for formal statistical comparison or ranking of the models.

### 4.11. Statistical Analysis

Statistical analyses were performed primarily in R. Within each model, differential gene expression was assessed using DESeq2, which models RNA-sequencing count data using a negative binomial generalized linear model. Doxorubicin-treated samples pooled across the available doses and time points were compared with the corresponding control samples. *p*-values were adjusted for multiple testing using the Benjamini–Hochberg procedure, and genes with a false discovery rate below 0.01 were considered differentially expressed.

WGCNA module–trait relationships were evaluated using the correlation-based statistical framework implemented in the WGCNA package. Overall treatment status, dose, and time were included as traits, and modules significantly associated with overall treatment status at a nominal *p*-value below 0.01 were selected for downstream analysis. Pathway enrichment was performed using Enrichr, and pathways with Benjamini–Hochberg-adjusted *p*-values below 0.05 were considered significant.

Principal component analysis was used descriptively to assess sample structure and treatment-related variation. Cross-model DEG overlap, directional concordance, Jaccard similarity, CTD coverage, and CTD directional concordance were treated as descriptive comparative measures. These measures were not used as formal tests of statistically significant differences between biological models.

## 5. Conclusions

We compared DOX-induced transcriptomic responses across human colonoids, mouse colonoids, and mouse colon to evaluate model concordance and distinguish conserved mechanisms from candidate human colonoid-specific responses requiring validation against human in vivo intestinal data. Despite limited cross-species gene overlap, pathway-level responses consistently converged on p53-linked DNA damage response, cell-cycle checkpoint regulation, DNA repair, and apoptosis, indicating a conserved toxicity core within model-specific responses. Mouse colonoids showed the highest DEG-level directional concordance with mouse colon, supporting their value for modeling epithelial components of the mouse in vivo response. CTD benchmarking provided additional toxicogenomic context but revealed only moderate directionality concordance, and highlighted GI-relevant genes that remain underrepresented in current curated resources. The comparative parallelogram approach identified conserved DOX-associated transcriptomic responses alongside notable model- and species-dependent differences. Human colonoids highlighted candidate human epithelial response features, including extrinsic apoptosis and p53-associated DNA damage and repair signals. Overall, these findings position colonoid-based transcriptomics as a useful mechanistic NAM for chemotherapy-induced intestinal toxicity assessment and support its potential contribution to reducing reliance on animal models. However, because colonoids lack vascular, immune, stromal, and systemic components, future validation against DOX-specific clinical or biopsy-derived intestinal data remains essential to define their predictive scope.

## Figures and Tables

**Table 1:** Shared DEGs across human colonoids, mouse colonoids, and mouse colon.

Gene Symbol	Gene Name	General Function
*INKA2*	Inka Box Actin Regulator 2	PAK4 inhibitor
*EPHX1*	Epoxide Hydrolase 1	Lipid metabolism
*PLK2*	Polo Like Kinase 2	Cell-cycle regulator
*MEP1B*	Meprin A Subunit Beta	Zinc metalloprotease
*ZMAT3*	Zinc Finger Matrin-Type 3	Apoptosis mediator
*AEN*	Apoptosis Enhancing Nuclease	Apoptosis mediator
*EI24*	Autophagy Associated Transmembrane Protein	Apoptosis mediator
*BAX*	BCL2 Associated X, Apoptosis Regulator	Apoptosis mediator
*CCND1*	Cyclin D1	Cell-cycle regulator
*GBA1*	Glucosylceramidase Beta 1	Lipid metabolism
*ITM2B*	Integral Membrane Protein 2B	Protease inhibitor
*LIPA*	Lipase A, Lysosomal Acid Type	Lipid metabolism
*POLR2H*	RNA Polymerase II, I and Subunit H	RNA polymerase
*PCNA*	Proliferating Cell Nuclear Antigen	DNA replication

## Data Availability

All RNA-seq data generated and/or analyzed in this study are available in the BioStudies database under accession numbers E-MTAB-16537 and E-MTAB-16565. The code used in this study is available at: https://github.com/saadlodhi0916/DOX-GI-Transcriptomics (accessed on 2 August 2026). Processed result tables supporting the analyses are provided in File S1.

## Supplementary Materials

The following supporting information can be downloaded at: https://www.mdpi.com/article/10.3390/ijms27167072/s1.

## Abbreviations

The following abbreviations are used in this manuscript:

AAALAC	Association for Assessment and Accreditation of Laboratory Animal Care
CIM	Chemotherapy-induced intestinal mucositis
CPM	Counts per million
CTD	Comparative Toxicogenomics Database
DDR	DNA damage response
DEGs	Differentially expressed genes
DMSO	Dimethyl sulfoxide
DOX	Doxorubicin
FDR	False discovery rate
GI	Gastrointestinal
GOIs	Genes of interest
NAMs	New Approach Methodologies
NGRA	Next-Generation Risk Assessment
PBPK	Physiologically based pharmacokinetic
PBS	Phosphate-buffered saline
PCA	Principal Component Analysis
PC1	Principal Component 1
R-ODAF	Omics Data Analysis Framework for Regulatory Application
RNA	Ribonucleic acid
RNA-seq	RNA sequencing
WGCNA	Weighted gene co-expression network analysis
