# Supplementary material for: Colonoid-Based Transcriptomics Reveals Conserved and Model-Specific Mechanisms of Doxorubicin-Induced Intestinal Toxicity
Source: Int J Mol Sci. 2026 Aug 7;27(16):7072. doi: 10.3390/ijms27167072 (PMC13513574; doi:10.3390/ijms27167072)
Supplement: Supplementary file 1 [file ijms-27-07072-s001.zip › ijms-4391091-supplementary.pdf]

Supplementary information to:

Colonoid-Based Transcriptomics Reveals Conserved and Model-Specific Mechanisms of Doxorubicin Induced Intestinal Toxicity

Saad Lodhi [1], * , Marcel Van Herwijnen [1] , Colette Kelly [2], Harvey Fowler-Williams [2], Carrie A. Duckworth [2], D. Mark Pritchard [2], Florian Caiment [1] , Theo M. de Kok [1], Marcha C.T. Verheijen [1], † and Danyel G.J. Jennen [1], †

1 Department of Translational Genomics, GROW - Research Institute for Oncology and Reproduction, Maastricht University, 6229 ER Maastricht, The Netherlands

2 Department of Molecular and Clinical Cancer Medicine, Institute of Systems, Molecular and Integrative Biology, University of Liverpool, Liverpool, L69 3GE, UK

* Correspondence: saad.lodhi@maastrichtuniversity.nl

†These authors contributed equally to this work.

Supplementary Figures and Tables

Table S1: Overview of dosing schemes, sampling time points, and numbers of animals (*in vivo*) or biological replicates (*in vitro*) across all experimental models.

**Mouse Colon (*in vivo*)**				
Timepoints	Controls	Dose (5mg/kg)	Dose (10 mg/kg)	
6 hours	3	4	4	
24 hours	3	4	4	
72 hours	3	6	6	
96 hours	3	6	6	
**Mouse Colon organoids (*in vitro*)**				
Timepoints	Controls	Concentration (0.1uM)	Concentration (1uM)	Concentration (10uM)
24 hours	3	3	3	3
48 hours	3	3	3	3
72 hours	3	3	3	3
**Human colon organoids (*in vitro*)**				
Timepoints	Controls	Concentration (1uM)	Concentration (10uM)	
24 hours	3	3	3	
48 hours	3	3	3	
72 hours	3	3	3	

Table S2. Summary of DEGs and module characteristics representing module trait correlation across mouse colon, colonoid and human colonoid datasets.

Dataset	Total DEGs	Module	Correlation	No. of Genes (per module)
Mouse colon	975	2	-0.43	106
		7	-0.47	81
		9	-0.33	29
		10	0.63	74
		13	0.32	505
		14	0.39	442
		15	0.4	1248
Mouse colonoid	1120	1	-0.46	364
		5	0.43	1809
		6	0.44	46
		8	-0.56	2145
		9	-0.4	36
		11	-0.4	206
		12	-0.56	65
		15	0.5	488
Human colonoid	564	4	-0.62	361
		5	-0.41	31
		8	0.42	177
		10	0.63	912

		12	0.56	32

Table S3. Summary of pathway enrichment results across models following doxorubicin exposure.

Model	DEG Pathways (padj < 0.05)	WGCNA Pathways (padj < 0.05)	Overlap
Human Colonoids	30	10	4
Mouse Colonoids	56	54	17
Mouse Colon	19	52	8

a

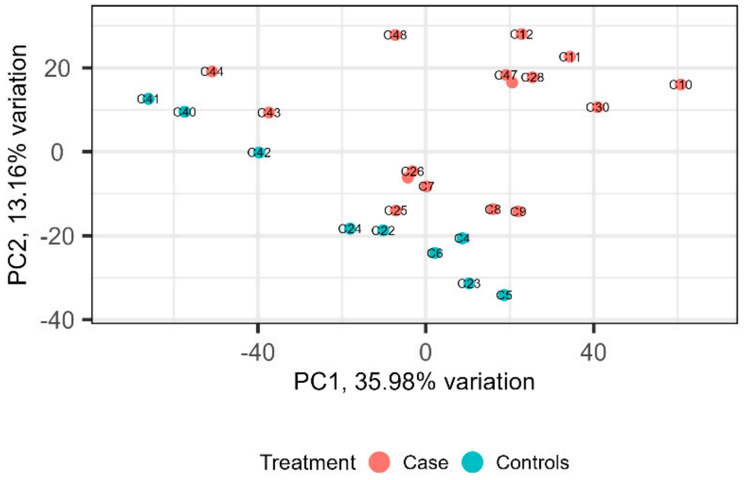

b

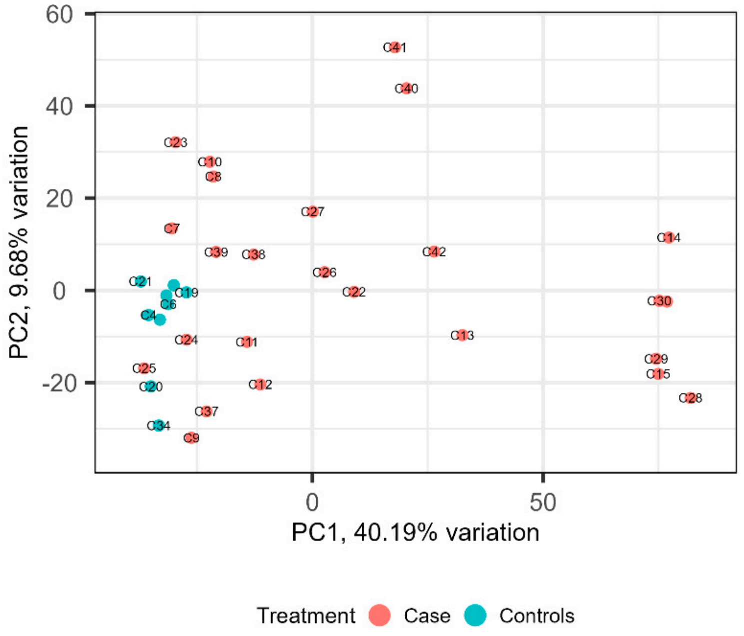

c

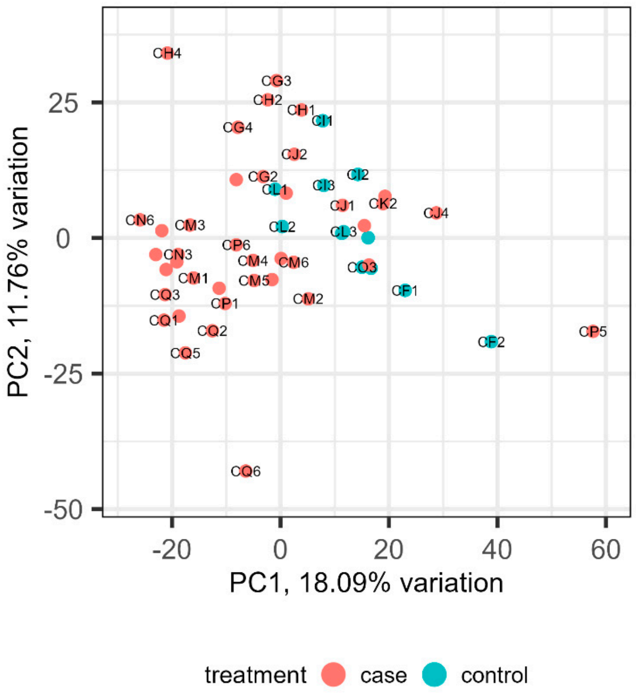

Figure S1. Principal component analysis (PCA) of RNA-seq expression profiles in each model. PCA plots are separately shown for (**a**) human colonoids, (**b**) mouse colonoids, and (**c**) mouse colon tissue. Each point represents one sample (sample IDs are labelled) and is coloured by treatment (DOX-treated case vs control). Axes show PC1 and PC2 with the percentage of variance explained.

a

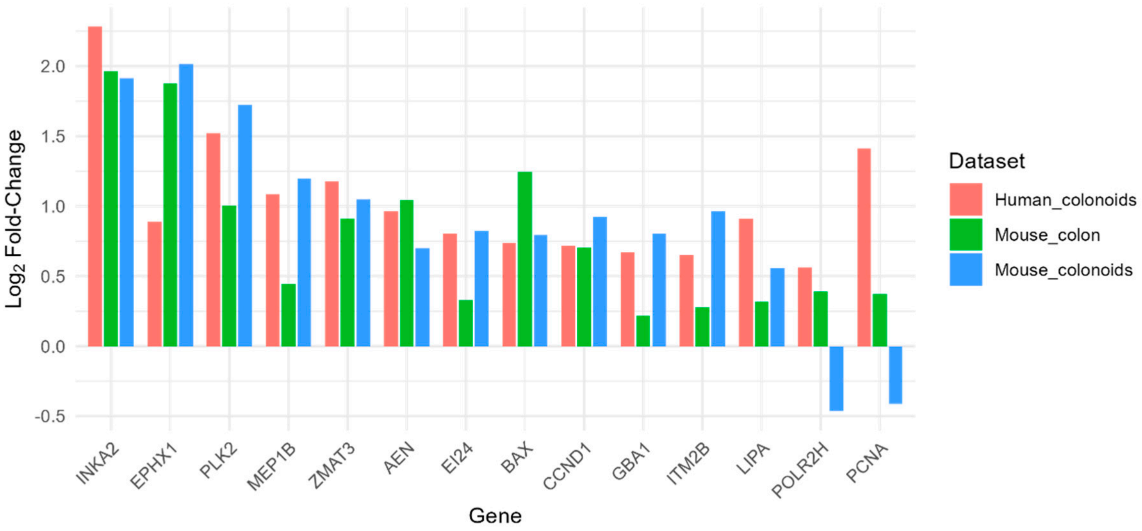

b

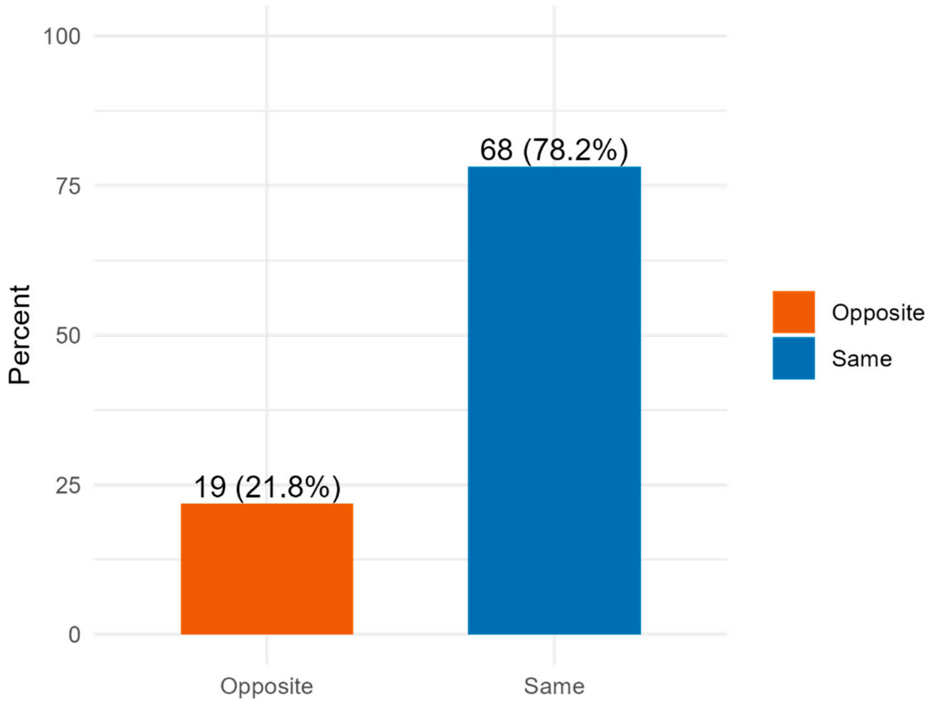
100
75
50
25
0
Percent
68 (78.2%)
19 (21.8%)
Opposite
Same
Opposite
Same

c

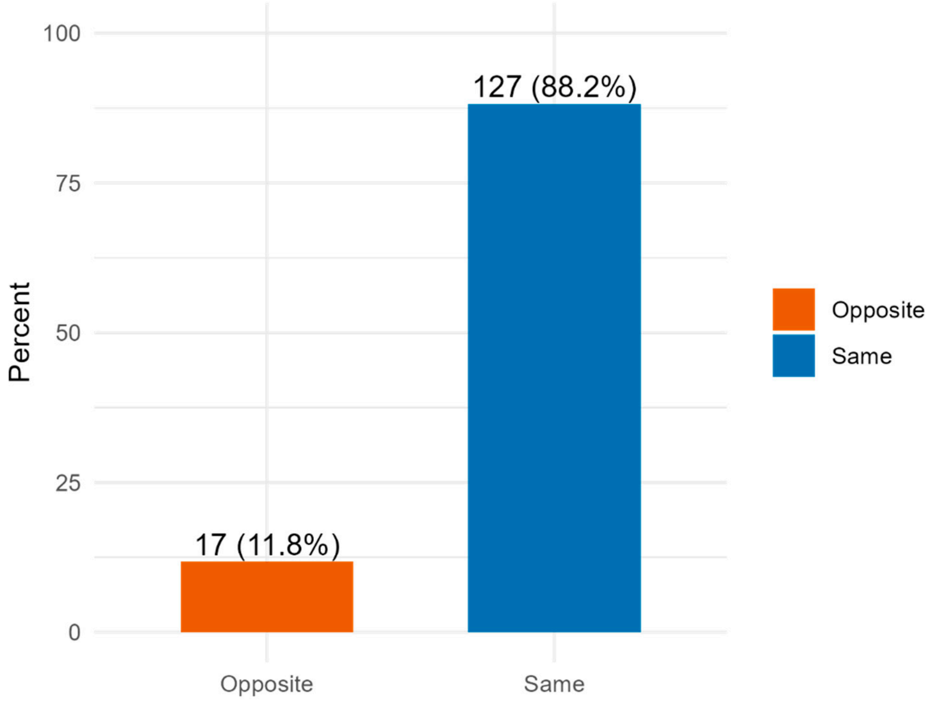
100
75
50
25
0
Percent
127 (88.2%)
17 (11.8%)
Opposite
Same
Opposite
Same

d

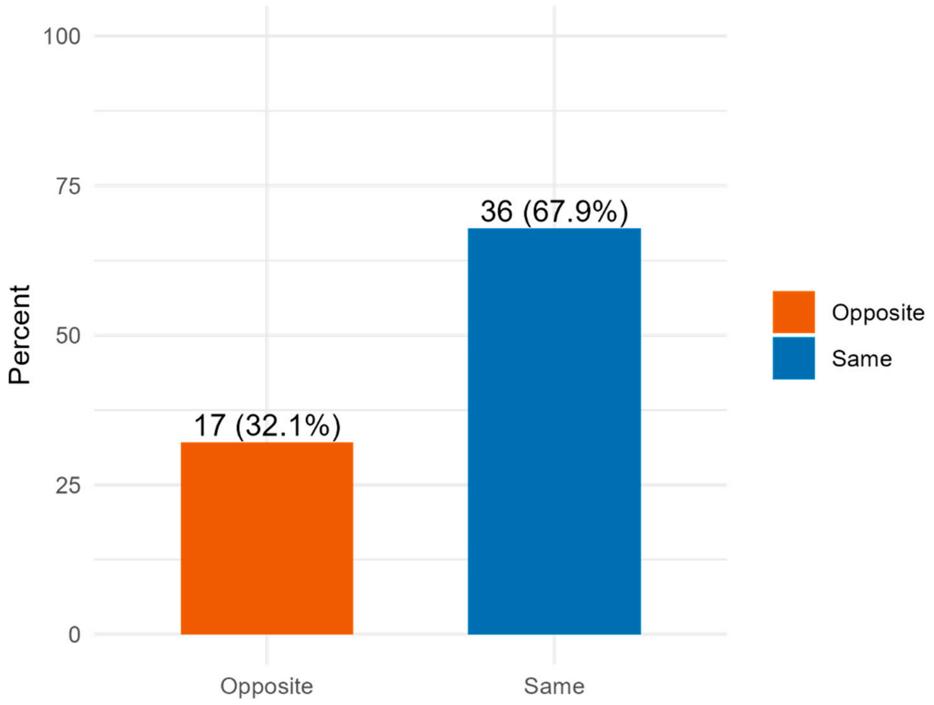

Figure S2. Gene expression directionality across all three datasets. (**a**) Represents Gene expression across the overlapping genes between the three datasets. (**b–d**) Percentage of gene expression directionality common and opposite between each pair .,i.e., (**b**) human vs mouse colonoids, (**c**) mouse colonoids vs mouse colon and (**d**) human colonoids vs mouse colon.

a

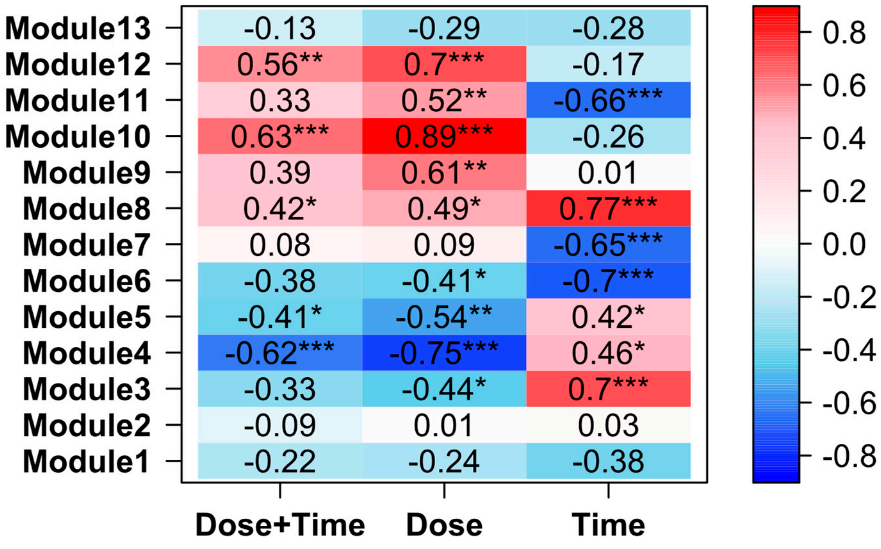

b

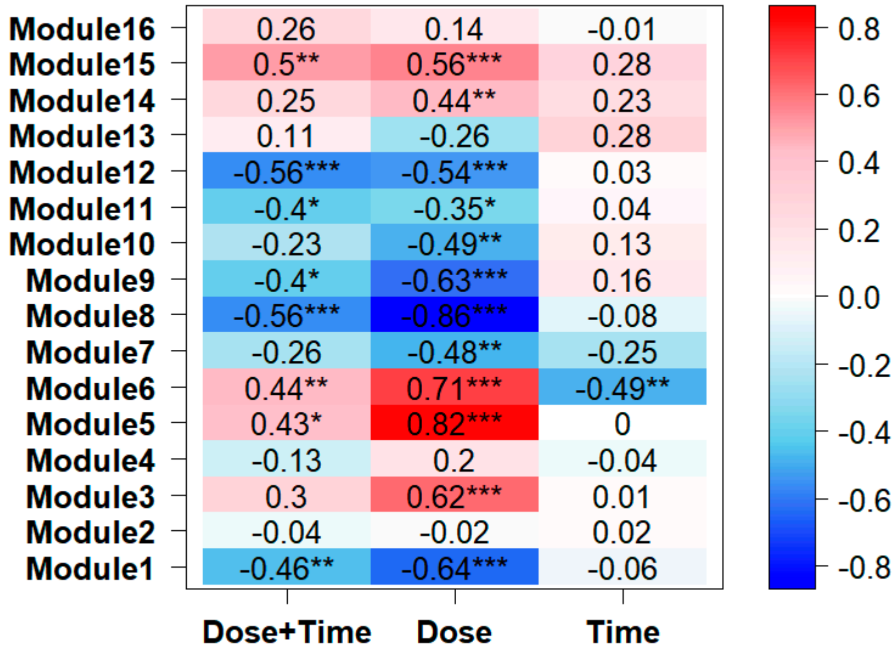

c

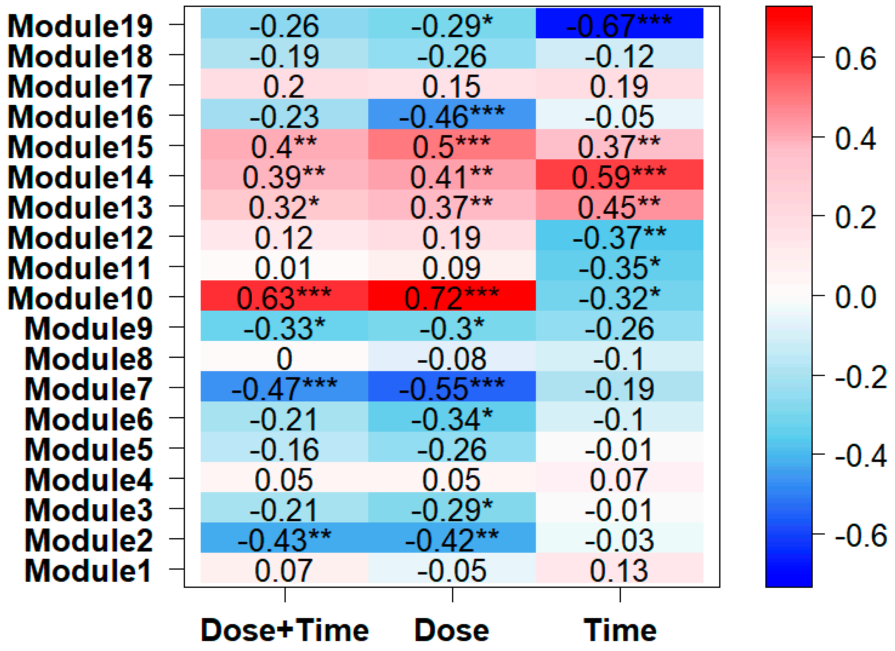

Figure S3. Module-Trait Relation identified by WGCNA in (**a**) human colonoids, (**b**) mouse colonoids and (**c**) mouse colon. Each heatmap shows the correlation between module eigengenes and the combined dose+time experimental traits. Color intensity reflects the strength and direction of correlation (red = positive, blue =negative) coefficients shown in each cell. Significance level is denoted by asterisks (* for p<0.05, ** for p<0.01, and *** for p<0.001).

a

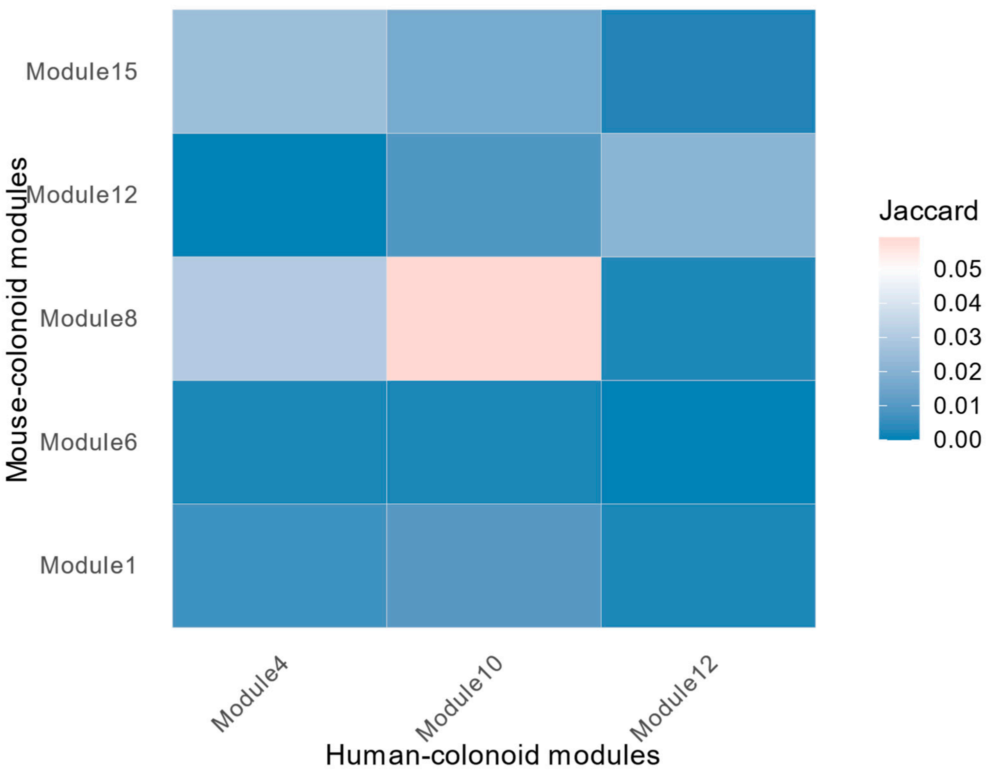
Module15
Module12
Module8
Module6
Module1
Mouse-colonoid modules
Module4
Module10
Module12
Human-colonoid modules
Jaccard
0.05
0.04
0.03
0.02
0.01
0.00

b

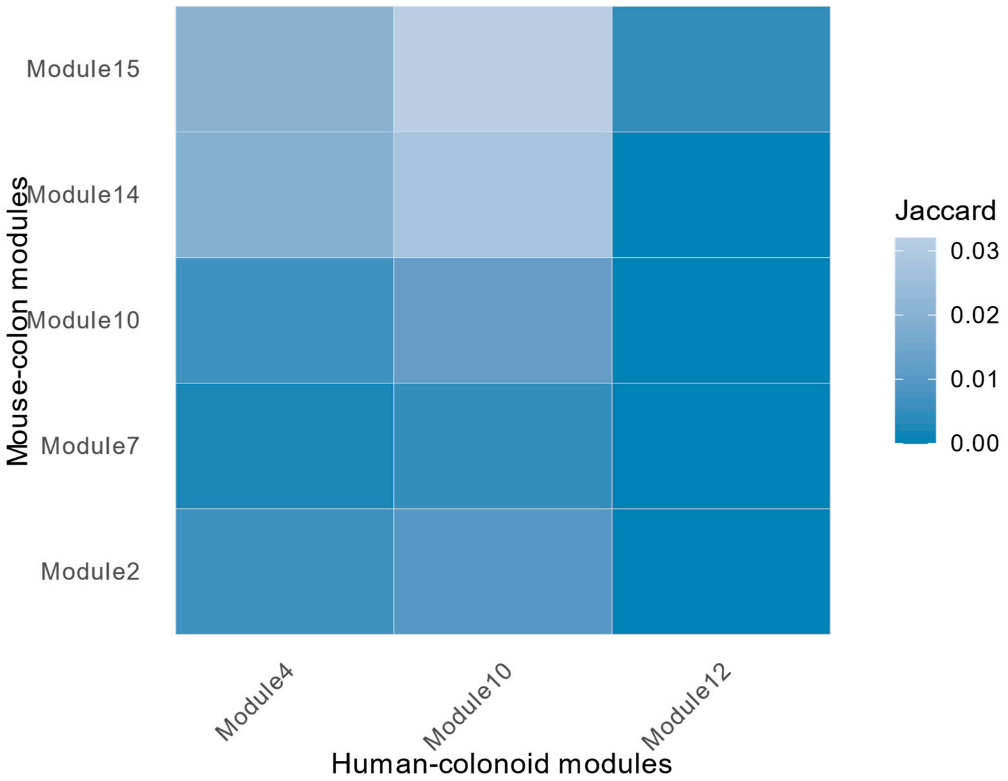
Module15
Module14
Module10
Module7
Module2
Mouse-colon modules
Module4
Module10
Module12
Human-colonoid modules
Jaccard
0.03
0.02
0.01
0.00

c

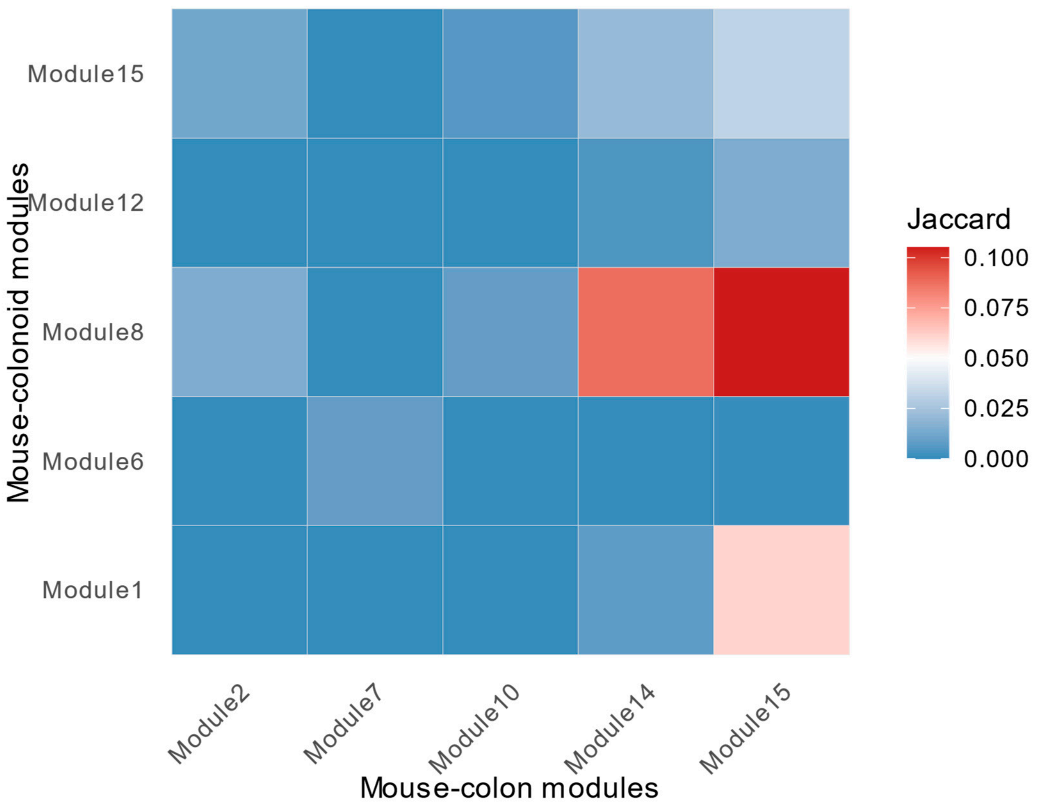

Figure S4. Cross-model similarity of significant WGCNA modules.

Heatmaps display pairwise Jaccard coefficients for the gene sets of significant modules. (**a**) Mouse colonoids vs human colonoids, (**b**) Mouse colon vs human colonoids, (**c**) Mouse colon vs mouse colonoids. Each panel represents the overlap between gene members of significant modules in two datasets. A continuous colour gradient runs from dark blue (J=0, no genes in common) to red (J=1, all genes in common). An intermediate value of J = 0.1indicates that approximately one tenth of the combined genes are shared by the two modules compared. A value of J=1 is not observed because the modules were derived independently in different datasets, so no two lists are identical.

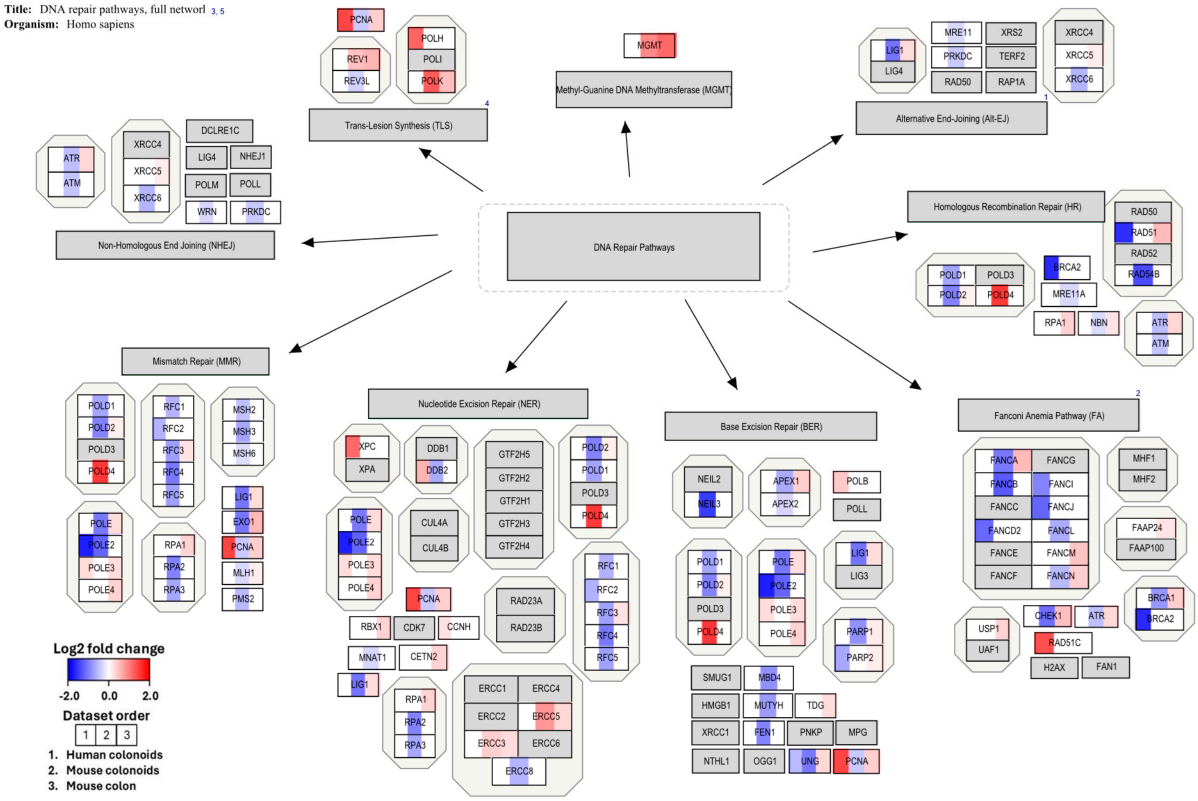

Figure S5. Gene mapping on the DNA Repair pathway in response to Doxorubicin across all three models. Each gene is depicted as a vertically segmented box colored by log_2(fold-change) (–2 = blue, 0 = white, +2 = red), with the left segment representing human colonoids, the middle segment mouse colonoids, and the right segment mouse colon.

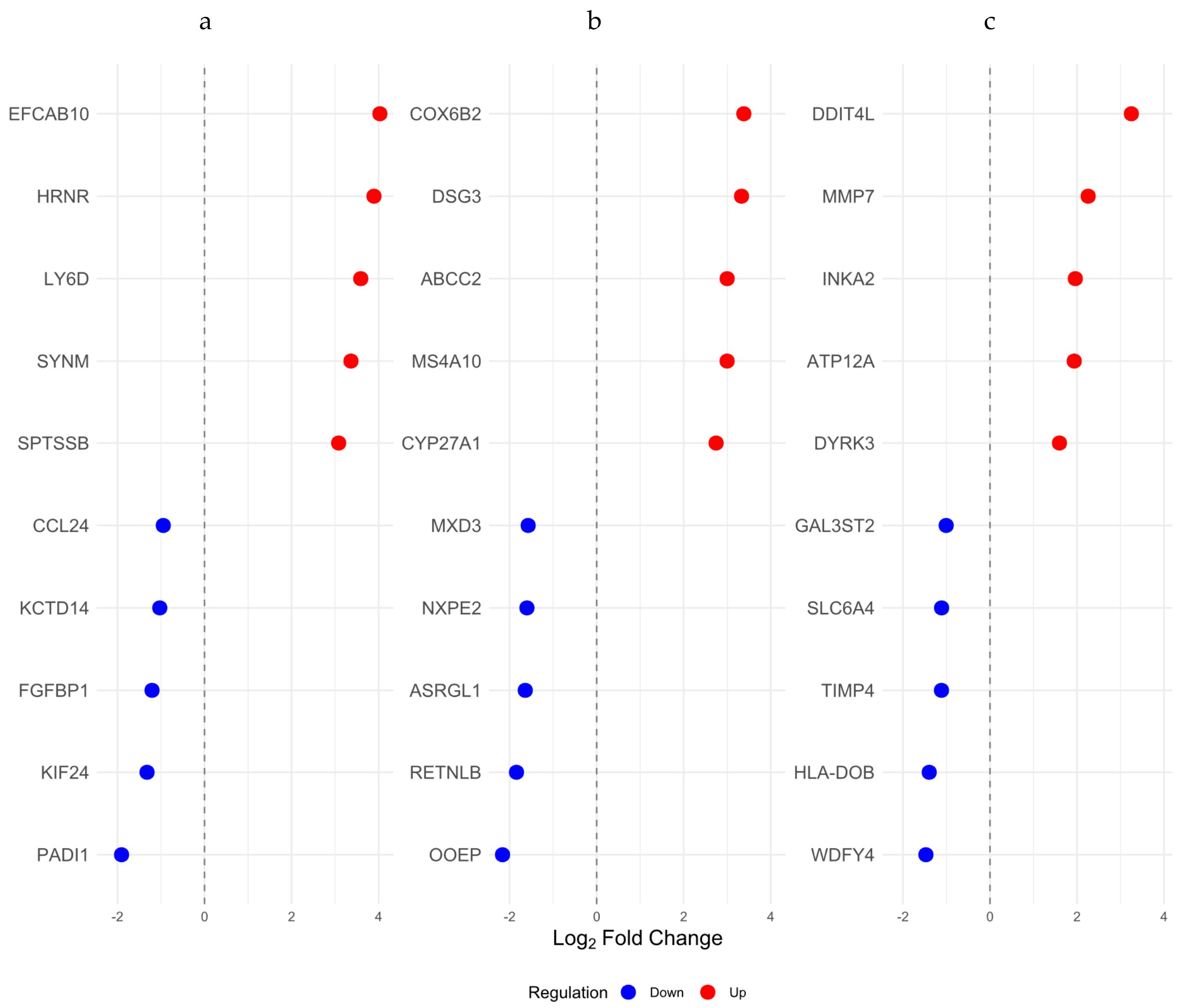

Figure S6. Top 5 up-and-downregulated non-CTD annotated genes in (**a**) Human colonoids, (**b**) Mouse colonoids (**c**) Mouse colon.
